# *Drosophila melanogaster* as a model to study autophagy in neurodegenerative diseases induced by proteinopathies

**DOI:** 10.3389/fnins.2023.1082047

**Published:** 2023-05-18

**Authors:** Stefania Santarelli, Chiara Londero, Alessia Soldano, Carlotta Candelaresi, Leonardo Todeschini, Luisa Vernizzi, Paola Bellosta

**Affiliations:** ^1^Department of Cellular, Computational and Integrative Biology (CiBiO), University of Trento, Trento, Italy; ^2^Department of Neuroscience, Scuola Internazionale Superiore di Studi Avanzati (SISSA), Trieste, Italy; ^3^Institute of Molecular Life Sciences, University of Zurich, Zürich, Switzerland; ^4^Department of Medicine, NYU Langone Medical Center, New York, NY, United States

**Keywords:** protein-aggregate, protein-misfolding, autophagy, neurodegeneration, proteinopathies, non-autonomous signaling, animal model, *Drosophila melanogaster*

## Abstract

Proteinopathies are a large group of neurodegenerative diseases caused by both genetic and sporadic mutations in particular genes which can lead to alterations of the protein structure and to the formation of aggregates, especially toxic for neurons. Autophagy is a key mechanism for clearing those aggregates and its function has been strongly associated with the ubiquitin-proteasome system (UPS), hence mutations in both pathways have been associated with the onset of neurodegenerative diseases, particularly those induced by protein misfolding and accumulation of aggregates. Many crucial discoveries regarding the molecular and cellular events underlying the role of autophagy in these diseases have come from studies using Drosophila models. Indeed, despite the physiological and morphological differences between the fly and the human brain, most of the biochemical and molecular aspects regulating protein homeostasis, including autophagy, are conserved between the two species.In this review, we will provide an overview of the most common neurodegenerative proteinopathies, which include PolyQ diseases (Huntington’s disease, Spinocerebellar ataxia 1, 2, and 3), Amyotrophic Lateral Sclerosis (C9orf72, SOD1, TDP-43, FUS), Alzheimer’s disease (APP, Tau) Parkinson’s disease (a-syn, parkin and PINK1, LRRK2) and prion diseases, highlighting the studies using Drosophila that have contributed to understanding the conserved mechanisms and elucidating the role of autophagy in these diseases.

## 1. Introduction

*Drosophila melanogaster* is an established model organism for developmental studies and due to the remarkable conservation of the signaling regulating autophagy, it has been used to better understand the relationship of this catabolic pathway with the genetic conditions that in humans are responsible of a class of neuronal diseases called proteinopathies (PPs). Autophagy is a key cellular pathway that, together with the ubiquitin-proteasome system (UPS), controls protein homeostasis by degrading misfolded proteins or exhausted organelles otherwise detrimental to the cells (Kocaturk and Gozuacik, 2018). Autophagy and UPS are closely linked, in fact protein ubiquitination is a key step for the cargo recognition by the autophagic receptors and alterations in one pathway may affect the activity of the other (Waite et al., 2022). Both pathways are crucial for cell survival particularly in neurons where their perturbation causes age-associated disorders including neurodegenerative diseases (Rai et al., 2022). In this review, we will illustrate the contribution of *Drosophila* studies to the understanding of the role of autophagy in PPs induced by mutations in genes responsible for the most common neurodegenerative diseases (summarized in Table 1). Furthermore, we will discuss how flies could be used to further improve our understanding of the mechanisms that control these diseases, particularly those that are linked to mutations in genes that are physiologically involved in the control of the autophagic-proteostatic pathway.

**Table 1:** *Drosophila* models of human proteinopathies discussed in this article, their principal mechanism, and components of pathways (modifiers) that can either suppress on enhance the toxic phenotype.

Disease	*Drosophila* model	Principal mechanism	Modifiers
Huntington’s disease (HD)	**HTT** Ectopic expression of *human HTT-Exon1-polyQ* (e.g., *HTT-Q93Q-ex1; HTT-Q72-GFP-ex1* Ectopic expression of human HTT-f*ull-length,* e.g.*, HTT-Q128-fl* (Romero et al., 2008)	The length of the CAG correlates with progressive motor decline and neuronal death (Marsh et al., 2000) Kinetic of aggregate formation (Weiss et al., 2012) Huntingtin role in autophagy (Ochaba et al., 2014) PolyQ oligomers forms “*de* novo” aggregates and increase their original size by directly using the endogenous prion-forming protein Rnq1 in its amyloid-like prion conformation (Gropp et al., 2022)	**Suppressor:** Endogenous *dhtt* (Zhang et al., 2010) TOR inhibition (Ravikumar et al., 2002; Sarkar et al., 2009) Chaperones (Sajjad et al., 2014; Pavel et al., 2016) Histone deacetylase (Pallos et al., 2008; Jia et al., 2012) Antioxidant pathway (Mason et al., 2013) deubiquitinating enzymes (Aron et al., 2018) Glutamine metabolism (Vernizzi et al., 2020) Rab5 (Ravikumar et al., 2008); PSA (Menzies et al., 2010) Compounds that target mHTT to autophagosomes (Li et al., 2019)
Spinocerebellar ataxias (SCAs)	**SCA1** Ectopic expression of human mutant *ATXN1*	Retinal degeneration and loss of interneurons projections (Fernandez-Funez et al., 2000) Reduced dendritic neurons arborization (Lee et al., 2011) Role of chaperones respect to polyQ containing proteins (Zhai et al., 2008)	**Suppressor:** TOR inhibition (Bouche et al., 2016) Rac-PAK pathway (Lee et al., 2011) CHIP and NMNAT (Zhai et al., 2008) Transglutaminases 5 (Lee et al., 2022b) Ataxin1 phosphorylation on Ser776 (Nitschke et al., 2021)
	**SCA2** Ectopic expression of human mutant *ATXN2*	Atx2 is involved in translational control (Satterfield and Pallanck, 2006) Aggregate formation and neuronal degeneration, critical for SCA3 (Lessing and Bonini, 2008)	**Enhancer:** Atx1 promotes Ataxin2 aggregation (Al-Ramahi et al., 2007)
	**SCA3** Ectopic expression of human mutant C-terminal *ATXN3*	Atx3 enhances the aggregation of the mutant *ATXN3* (Johnson et al., 2019) Catalytical activity of Atx3 is necessary for its autoprotective role (Warrick et al., 2005) Mutant *ATXN3* expression leads to abnormal eye morphology and motility defects (Warrick et al., 1998)	**Suppressor:** Functional *ATXN3* deubiquitination activity (Warrick et al., 2005) Ubiquitin ligases as Cullins and Praja1 (Chen et al., 2019; Ghosh et al., 2021) Antioxidant drugs (Wu et al., 2017) **Enhancer:** Hsc70-4 (Johnson et al., 2020)
Amyotrophic lateral sclerosis (ALS)	***C9orf72*** *C9orf72* transgenic flies carrying different length of ‘pure’ GGGGCC sequence contained in the human gene (e.g., *GGGGCCx36* or *GGGGCCx103*) Expression of polypeptides containing GA, GP, GR or PA repeats (that may carry GFP or FLAG tags)	Toxicity of different numbers of GGGGCC repeats and formation of RNA foci (Mori et al., 2013; Xu et al., 2013; Mizielinska et al., 2014) Polypeptides containing GR and PR repeats are the most toxic role of *C9orf72* in autophagy (Wen et al., 2014; Lee et al., 2016; Cleary et al., 2018; Cunningham et al., 2020)	**Suppressor:** overexpression of SIGMAR1 (Lee et al., 2020a)	
	**SOD1** *SOD1* LOF mutations in *Drosophila*’s *SOD1* gene or *null dsod* mutants Expression of human mutant *SOD1*	SOD1 is involved in protein misfolding, and it is necessary for neuronal health (Şahin et al., 2017; Agudelo et al., 2020) SOD1 was found in a complex with Atg9/Beclin1 to control P62/SQSTM1 accumulation (Nassif et al., 2014) Mutations in *SOD1* lead to mitochondrial impairments and non-autonomous signaling from glial to neurons and in MNs (Tafuri et al., 2015)	**Enhancer:** DUB-USP7 (Zhang et al., 2020) L3MBTL1 and SETD8 (Lu et al., 2019) USP7 which reduces SMAD2/TGF-β pathway (Zhang et al., 2020)	
	**TDP-43** TDP-43 expression of human TDP-43 or its mutant form (TDP-43-M337V, with unfunctional NLS) LOF variants of *Drosophila*’s *TBPH*	TBPH-null mutant is semi-lethal in flies (Donde et al., 2020).	**Suppressor:** Atg7 (Donde et al., 2020) HSP67Bc (Crippa et al., 2016). HEXA-018 treatment (Lee et al., 2021)	
	**FUS** *FUS* expression of mutant forms of human *FUS* (e.g., FUS-518 K, -R521C or -R521H)	Expression of wild-type FUS and of ALS-related FUS mutations triggers the accumulation of toxic aggregates that inhibits autophagy (Brunet et al., 2021) FUS and TDP-43 interact to induce neurodegeneration (Lanson et al., 2011)	**Suppressor:** Mask promotes autophagy by expression of the proton-pumping vacuolar (V)-type ATPases (Zhu et al., 2017) Inhibition of PI3K/AKT/TOR ameliorate P525L-FUS mutation due to induction of autophagy (Marrone et al., 2018). Glutathionylation by Glutathione transferase omega 2 promotes FUS degradation (Cha et al., 2022)
Alzheimer’s disease (AD)	**APP** Expression of human Aβ peptides or human AD-associated genes (e.g., human *APP* and *BACE1*)	Lowering the expression of *Atg1*, *Atg8a* and *Atg18*, enhances the neuronal toxicity caused by Aβ expression Dysfunctional AEL (autophagy-lysosomal-endosome vesicles) induces amyloid-plaque formation Ectopic APP expression leads to aberrant autophagy (Ling et al., 2014; Omata et al., 2014; Zhuang et al., 2020; Tsakiri et al., 2021; Zhu et al., 2022)	**Suppressor:** NMNAT (Zhu et al., 2022). Trx80 (Gerenu et al., 2021) **Enhancer:** Lowering of autophagy-related genes (Omata et al., 2014)
	**Tau** modulation of the expression of full-length human *Tau* gene	Insights in Tau toxicity and autophagy (Chatterjee et al., 2019; Subramanian et al., 2021)	**Suppressor:** Ataxin3 deubiquitinase activity (Bilen and Bonini, 2007) Calpain silencing (Menzies et al., 2015) CG11070 (Subramanian et al., 2021) Decrease in PTK2 expression (Lee et al., 2022a) **Enhancer:** Insulin signaling (Chatterjee et al., 2019)
Parkinson’s disease (PD)	**α-synuclein** expression of mutant human *SCNA* gene (e.g., α-syn-A30P; α-syn-A53T)	Impairments in the autophagic flux and in mitophagy (Sarkar et al., 2021).	**Suppressor:** Spermidine (Buttner et al., 2014) LAMP2A (Issa et al., 2018) dDOR (Dinh et al., 2021) knockdown of inositol-requiring enzyme 1 (IRE1) and Atg7 (Yan et al., 2019)	
	**Parkin/PINK1** *Parkin/PINK1* null mutants for *Drosophila*’s *parkin* and *pink* Ectopic expression of mutant human *parkin* and *PINK*	Relevance of Parkin and Pink in mitophagy molecular process (Greene et al., 2003; Ishihara-Paul et al., 2008; Hewitt and Whitworth, 2017) The age-related increase in mitophagy depends on the interaction of parkin and Pink with UPS-15 and − 30 (Cornelissen et al., 2018) Mitochondria phenotype in *parkin* null mutants recapitulates autophagy inhibition in loss of *Atg7* (Vincow et al., 2013)	**Suppressor:** BNIP3L (Gao et al., 2015) Downregulation of ANKHD1 (Zhu et al., 2015)	
	**LRRK2** LOF *dLRRK* models Expression of human *LRRK2* mutants (e.g., LRRK2-G2019S)	Overexpression of human mutant LRRK2 induces defects in autophagy (Hewitt and Whitworth, 2017; Seegobin et al., 2020; Dodson et al., 2014; Steger et al., 2016; Bellucci et al., 2022) *dLRRK* is essential for functional autophagic flux and vesicle trafficking, also at the synaptic level (Ng et al., 2012; Steger et al., 2016; Bellucci et al., 2022)	**Suppressor:** Expression of parkin and AMPK activation (Ng et al., 2012)
Prion Diseases (PrD)	**PrP** Expression of mutant PrP forms of human or murine genes (e.g., Prp-PG14, Prp-P101L, PrP-3F4, PrP-M129, PrP-V129 and from human diseases PrP-GSS, FFI and CJD)	Insights in PrP misfolding process (Thackray et al., 2020) Reversibility of the human PrP-GSS phenotype using inducible system (Murali et al., 2014) Transferability of the pathology between flies or from mice to flies (Thackray et al., 2017) Toxicity of different aminoacids substitutions in PrP protein (Myers et al., 2022) Perturbation of TOR signaling-related and cell-cycle related genes expression (Thackray et al., 2020) Co-localization of PrP^Sc^ in large aggregates with p62/SQSTM1 (Homma et al., 2014)	**Suppressor:** 4E-BP activity suppresses human PrP-M129 and PrP-V129 mutations (Myers et al., 2022)

Their link to autophagy is also outlined.

## 2. *Drosophila* versus human brain

*Drosophila* ‘s studies played a crucial role in understanding brain function and development, leading to significant advances in neuroscience. *Drosophila*’s brain contains about 200,000 neurons, which are quite similar to mammalian neurons in terms of electrophysiological properties, and form a complex network of interactions recently mapped at high resolution (Zheng et al., 2018), and glial cells that represents 5–10% of the total cell population within the central nervous system (CNS). The CNS is composed of multiple specific and distinct brain compartments (Ito et al., 2014) that are interconnected and synergistically cooperating to control complex behaviors, such as learning, flight control, courtship, grooming, and memory-driven behaviors. Despite the small size, the overall brain organization and regional division shows fundamental similarities to the network structure of the mammalian brain. Indeed, the neural organization underlying primitive functions, such as the perception of odors, taste, vision, sound, gravity, and the circuits regulating feeding and satiety are very similar to those in humans (Tsubouchi et al., 2017; Jayakumar and Hasan, 2018). *Drosophila* CNS can be divided into two histological regions: the neuronal cell cortex, where all the neurons cell bodies are located, and the neuropil, where axons and dendrites project. This represents a major difference with vertebrates, where the cell bodies are in different areas of the brain depending on the circuit that they are regulating. In addition, in the fruit fly, the lateral connections between neighboring projection-neurons of the same compartment are made not only through interneurons (Liou et al., 2018) but also through dendro-dendritic synapses, which are rare or absent in most vertebrate neuropiles. In terms of morphology, while in vertebrates most neurons are multipolar, in invertebrates there are mostly unipolar (Rolls, 2011; Shah et al., 2016; Smarandache-Wellmann, 2016). Despite these differences, both vertebrate and invertebrate neuronal circuits are plastic which means that their structure and physiology are modified in response to stimuli, both extrinsic and intrinsic, during development and in adult life (Holtmaat and Svoboda, 2009; Lin et al., 2013; Peretti et al., 2015). The electrophysiological properties of the fruit fly neurons are also very similar to those in mammals. Their firing activity depends on Na^+^ and K^+^ fluxes that affect the membrane potential, and they communicate using vesicle release of conserved neurotransmitters (i.e., acetylcholine, GABA, glutamate) and neuromodulators (i.e., biogenic amines and neuropeptides) at the synapses. In *Drosophila* there are several classes of glia, mostly classified based on their morphology function and association with neurons. The perineural glia (PNG), which surrounds the central nervous system, is required to filter nutrients, while the sub-perineural glia (SPG) forms the septate junctions and together form the blood–brain barrier (BBB). This is different than in humans, where the BBB is composed of astrocytes and microglia, however also in *Drosophila* the BBB prevents paracellular diffusion and controls the influx and efflux of soluble molecules (Hindle and Bainton, 2014; Babatz et al., 2018; Kim et al., 2020). Within the CNS there are other classes of glia cells such as the cortex glia, associated with the neuronal cell bodies, unsheathing glia, that surrounds neuropils and astrocytes that associates closely with the synaptic neuropils and support neurons, similarly to those of the mammalian glia. These classes of glia are abundant and are involved in nervous system development, circuit assembly, synaptic plasticity and neurons support (Freeman, 2015).

## 3. *Drosophila’s* tools to study proteinopathies (PPs)

**Figure 1:**
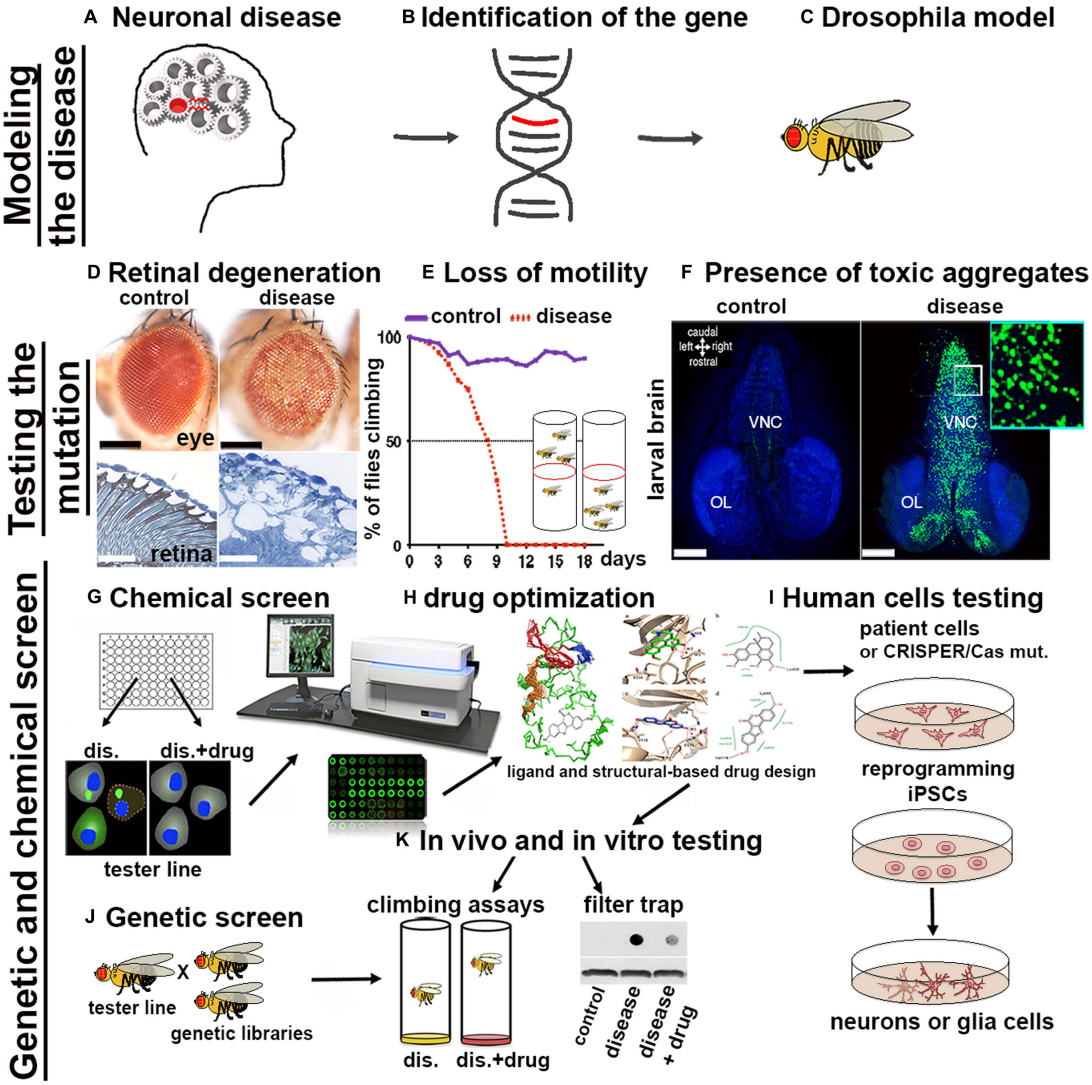
Scheme of a pipeline to characterize genes associated with proteinopathies and to perform High-throughput Screens with small chemical compounds, to develop new therapeutical strategies in humans. **(A–C)** From the identification of a gene related to a human disease to the generation of the transformants carrying the GOI. **(D–F)** The effect of the mutant-disease genes can be tested at cellular and behavioral levels. **(D)** For example, the expression of *exon1* of the human mutant *Huntingtin* containing 93-CAGs (*HTTQ93*) in the retina using the *GMR-Gal4* promoter leads to loss of the pigmentation in the ommatidia of the compound eye (as seen in the upper image obtained using a stereo microscope) and to retinal degeneration accompanied by defects in tissue morphology and neuronal death [outlined by the white spot of missing tissues visible by transmission electron microscopy (TEM) showed in the images below; (Vernizzi et al, 2020)]. **(E)** Expression of mutant HTT in neurons using *ELAV-Gal4* induces neuronal defects that can be indirectly quantified by measuring the decline over time of the animal motility (negative geotaxis assay). **(F)** Using the *Elav promoter* we can express the human *HTT exon-1 with 97 CAGs* as a fusion protein with GFP *(HTT-GFP)*, and show the formation of HTT-GFP aggregates in larval neurons already at 48-72 hours of age. Photo in panel **F**, to the right is shown a larval brain of *Elav-LexOP-HTTGFP-LexA larvae at 72 hrs AEL*, control is to the left. BLUE stains the nuclei (inset 63x). **(G–K)** Potential pipeline for a High throughput screen (HTS) to identify drugs that reduce the formation of toxic aggregates. **(G)** *Drosophila* cells, induced to express the *HTTGFP* construct are cultured in medium containing small chemical compounds (libraries); analysis of the changes in GFP expression can be quantified using a microplate reader (TECAN). Compounds capable of reducing GFP expression will highlight potential pathways that could be involved in the reduction of mHTT aggregates. **(J)** *Drosophila* HD models can be used to perform genetic screens to analyze *in vivo* the expression of components of these pathways; **(K)** *Drosophila* HD models will be fed with the small compounds identified to analyze their effect in ameliorating animal motility or in reducing the size of mHTT aggregates; analyzed directly by immunofluorescence in the brain or by filter-trap assays using either organs or from whole animals. **(H)** For better performance, chemical drugs can be modified using ligand and structure-based drug design to improve their characteristics and then they can be tested again *in vivo* in *Drosophila* HD models such as in K. **(I)** Finally, the candidate drugs will be tested using human cells differentiated to iPSCs and then to neurons of glia, or directing to neurons iNs (Hong and Do, 2019) starting from cells of patients or from cells from healthy donors in which the specific mutation is introduced using the CRISPR/CAS9 technique.

The availability of a wide variety of transgenic strains, advanced genetic tools and databases have enabled the rapid development of *Drosophila* models for nearly 75% of human neuronal diseases including proteinopathies PPs (Ugur et al., 2016). The simplicity of fly’s brain architecture and the highly conserved function of genes involved in neuronal development, highlights another advantage for its use. Furthermore, *Drosophila*’s genome generally harbors only one orthologue of the human counterpart therefore, mutation of a single gene generally leads to loss-of-function phenotypes, without redundant effects due to the presence of compensatory paralogues. The availability of unique and advanced genetic techniques allows for the rapid generation of transgenes in which the manipulation of the gene of interest (GOI) is performed in a short time and with unique precision in specific tissues and organs. The most common approach used is based on the yeast derived UAS/Gal4 system, in which a line carrying a tissue specific promoter fused with the transactivating domain of Gal4, is crossed with another line carrying the GOI (overexpression or its RNAi) cloned under the control of the UAS (Upstream regulating sequence). In the progeny, the binding of Gal4 to the UAS sequence will express or reduce the GOI in the tissue of interest (Brand and Perrimon, 1993). If manipulation of the GOI induces lethality, tissue expression control can be achieved by co-expression of the temperature sensitive inhibitor GAL80^ts^, which acts as a transcriptional repressor of Gal4 at its permissive temperature, or by the Gene-Switch system which relies on a modified version of Gal4 which can be temporally induced by the administration of the drug RU486 (Walters et al., 2019). Variants of the UAS/Gal4 system, such as LexA/Op and QUAS, can be used to drive the expression of different transgenes concurrently with the UAS/Gal4. This is particularly useful for example to study the presence of non-autonomous signals between different organs, tissues or cells *in vivo* (Potter et al., 2010). Additionally, loss-of-function mutations or overexpression of genes can be easily obtained using CRISPR/Cas9 technology and commercially available sgRNA lines deposited at the Bloomington Stock Center (Ewen-Campen et al., 2017; Zirin et al., 2022). More recently, novel optogenetic techniques have been successfully developed to study protein misfolding *in vivo* in the brain using *Drosophila* models of Alzheimer’s disease (AD), Parkinson’s disease (PD), and TDP-43/ALS (Lim et al., 2021). The morphological defects induced by the expression of genes responsible for human PPs (Figures 1A,B) can be easily analyzed after their expression in *Drosophila* (Figure 1C) using *ex vivo* dissection of the entire larval or in adult-fly brains, using different techniques ranging from immunofluorescence to super resolution microscopy (SEM or TEM; Figure 1D). Furthermore, motor defects caused by neuronal degeneration can be characterized by analyzing larval motility defects or by analyzing the decline of negative geotaxis in adults, which is the natural ability of flies to climb against gravity (Figure 1E). PPs are characterized by the formation of protein aggregates, which can be visualized by immunofluorescence using specific antibodies, or with the expression of fusion proteins where the gene of interest is fused with fluorochromes such as GFP or RFP (Figure 1F). Biochemical assays, using techniques such as western blot or filter-trap analysis, can show variations in molecular weight and the presence of large insoluble aggregates (Figure 1K). Cell death or induced inflammation at sites of aggregate formation can be studied directly by immunofluorescence assays using appropriate markers (e.g., cleaved caspase, TUNEL assay, or the presence of secreted immune-modulators, like eiger/TNFα or SPARK). Finally, one of the great advantages of using fruit flies as a model for PPs is that the onset of these diseases is relatively rapid, for example the expression of human mutant forms of Huntingtin *HTTQ97GFP*, in neurons leads to the formation of aggregates which are already visible within 48–72 h of larval development (Figure 1F). This is extremely advantageous since it allows to perform genetic or chemical screening to identify in a short time genes or drugs that reduce the formation of the aggregates (Figures 1G,J). These inhibitors can be chemically optimized (Figure 1H) to improve their qualities and finally be tested on human cells derived from patients (Figure 1I). However, questions remain as to why flies develop aggregates so early in their development, compared to vertebrates. One hypothesis is because flies lack a more complex, adaptive immune system that could control the onset of these diseases as it does in other model systems. In summary, the possibility to target specific mutations into subpopulation of neurons or glia and the ability to rapidly observe their effect cell autonomously or across the neural networks, combined with the short life cycle and the propensity to produce many offspring confirms *Drosophila* as an excellent model for studying human diseases including proteinopathies.

## 4. Mechanism of autophagy

**Figure 2:**
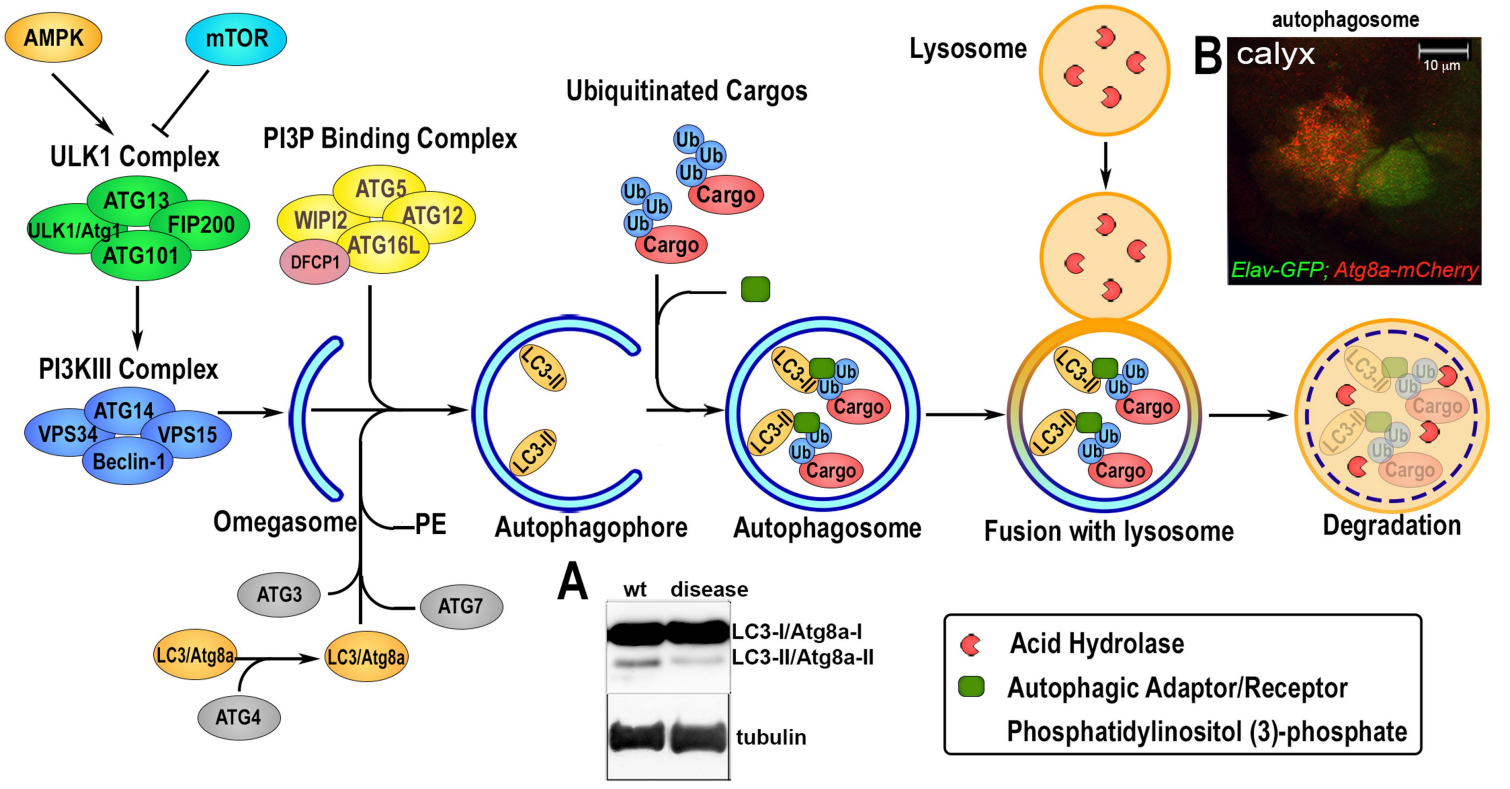
Overview of the autophagic flux. The induction of the autophagic process is regulated upstream by 5†’-AMP-activated Protein Kinase (AMPK) and Target of Rapamycin (TOR), that modulate the activation of the Unc51-like Kinase 1 (ULK1/Atg1) via phosphorylation and the formation of the initiator the ULK1-Complex composed by ULK1, Autophagy-related protein 13 (ATG13), ATG101 and FIP200. Upon activation, ULK1/Atg1 Complex in turn activates via phosphorylation the phosphatidylinositol-3-kinase III (PI3KIII) Complex, which comprises vacuolar protein sorting 34 (VPS34), VPS15, ATG14 and Beclin-1. This allows its translocation on the ER membrane and the production of an isolation membrane enriched in phosphatidylinositol (3)-phosphate (PI3P). PI3P induces the recruitment of the PI3P Binding Complex, consisting of ATG5, ATG12, ATG16L and WD Repeat Domain, Phosphoinositide Interacting 2 protein (WIPI2), on the growing autophagophore (omegasome) and the Double FYVE-containing protein 1 (*DFCP1*). This process enhances the ATG3-mediated binding of Microtubule-associated protein 1A/1B-light chain 3 (LC3) to phosphatidylethanolamine (PE) on the autophagosomal membrane where is lipidated. This leads to its cleavage from LC3-I/ATG8a-I to LC3-II/ATG8-II, which is considered a feature of an active autophagic flux and can be visualized and quantified by western blot analysis (panel **A**). Ubiquitin-tagged proteins are recognized by specific autophagic adaptors/receptors, such as p62/SQSTM1/Ref2(P), with a mechanism that is selective for each different organelles or cellular structure called selective autophagy. The cargo receptors bind LC3/ATG8a and transport the cargo into the autophagophore where its content is hydrolyzed upon fusion with lysosome. Autophagosome formation can be visualized *in vivo* by ectopic expression of LC3/Atg8a fused to GFP or mCherry fluorescent proteins that form small “puncta” on the autophagosome membrane, and fluorescence can be quantified using imaging processing programs. Panel **B** shows the *UAS-Atg8a-mCherry* staining pattern under physiological conditions analyzed in the calyx region of *Drosophila* larval brain, where neuronal cells are visualized by expressing *UAS-GFP* using the *ELAV-Gal4* promoter. Co-localization of mCherry puntae within GFP is used to quantify the presence of the autophagosome in neurons.

Autophagy is a conserved mechanism with different specific catabolic functions in different tissues and cells. For example, in conditions of nutrient deficiency it represents a survival mechanism that generates amino acids and bioenergetic substrates to allow cell survival. However, autophagy is also used to eliminate toxic debris and exhausted organelles to maintain cellular clearance, particularly relevant in aging neurons, where physiological reduction in autophagy can cause their premature loss. Autophagy can occur in three different forms: microautophagy that is mediated by small cargo-containing vesicles on the lysosomal membrane; chaperone-mediated autophagy (CMA), in which the chaperone Hsc70, a member of the Hsp70 heat shock protein family, recognizes cargo proteins containing KFERQ-like motifs and delivers them to the lysosomes via the receptor lysosome-associated membrane protein 2A (LAMP2A); and the most studied macroautophagy herein referred as autophagy, mediated by a subset of ATG proteins encoded by the *ATG* gene-family, originally identified in yeast in response to nutrient starvation (Fleming et al., 2022). The product of the *ATGs* genes is responsible for the initial formation of the phagophore that envelops cytoplasmic cargoes in a double membrane (autophagosomes) which subsequently fuses with the lysosomes for the degradation of the cargo by hydrolases. Due to the specificity of different cargoes, autophagy is now studied and classified into more specific pathways (selective autophagy) such as: lipophagy (lipid droplets), ERphagy (endoplasmic reticulum), mitophagy (mitochondria), pexophagy (peroxisomes), aggrephagy (protein aggregates) and xenophagy (bacteria and viruses; Galluzzi et al., 2017). An important step in selective autophagy is the interaction of the ubiquitinated cargo-proteins with autophagic receptors, such as p62/SQSTM1 (Ref2(P) in flies) that by binding LC3 (ATG8a in flies) via the LIRs (LC3-interacting regions) sequences, recruits cargoes into the autophagosome for degradation. The amino acids produced in the lysosome by hydrolysis of the cargo can directly re-activate the TORC1 complex, located on the lysosomal membrane, thus blocking the autophagic flux (Abu-Remaileh et al., 2017; Wyant et al., 2017). Indeed, TOR kinase, which is part of the TORC1 complex, acts as negative regulator of autophagy by phosphorylating specific sites of the Ser/Tre kinase ULK1/2 (ATG1 in flies) thus destabilizing the initiator complex of the autophagy process that is composed by ATG13, ATG101, FIP200 (Figure 2). This is an essential step for the phagophore assembly since this complex activates local phosphatidylinositol-3-phosphate (PI3P) production at membrane structures called omegasome, that recruits the WIPI2 (WD repeat domain phosphoinositide-interacting proteins) and DFCP1 (zinc-finger FYVE domain-containing protein 1). These proteins are important for the recruitment of the ATG6L1-ATG5-ATG12 complex, responsible for the ATG3-mediated conjugation of LC3 proteins to membrane phosphatidylethanolamines (PEs) to form the membrane double bond. In this process, LC3-I is cleaved at the membrane and converted into LC3-II, process that is also used as a signature of autophagic flux efficiency and typically visualized in western blot assays (Figure 2A). Ectopic expression of LC3 fused to a Fluorescent Proteins is used as a marker of the autophagosome and to quantify the autophagic vesicles (in Figure 2B is shown *Atg8a-mCherry* expressed in neurons of the calyx together with *GFP* using the *Elav-Gal4* promoter). Autophagy is also regulated by AMPK, a kinase that is activated by cellular stress and low levels of ATP. AMPK phosphorylates ULK1/2 (ATG1 in flies) at sites different than those targets of TORC1. This promotes the formation of the initiator complex of autophagy (Figure 2; Kim et al., 2011). Therefore, molecules capable of inducing basal autophagy, such as rapamycin (a potent TORC1 inhibitor), or metformin (which activates AMPK) represent important therapeutic targets in proteinopathies. The use of simple animal models such as *Drosophila* was particularly relevant to the discovery and initial characterization of such molecules.

### 4.1. *Drosophila*’s autophagy a link to neuronal degeneration

Most of the steps in the autophagy pathway were first identified and characterized in the *Drosophila* fat body, where there is a physiological induction of autophagy during metamorphosis to allow the animal to survive starvation (Rusten et al., 2004; Scott et al., 2004). Furthermore, because of the big size of the fat cells (50 μm), the fat body was used to better visualize the autophagosome maturation using ATG8a conjugated with mCherry or GFP (Figure 1B). Subsequently, genetic studies have delineated the important control of autophagy in longevity (Maruzs et al., 2019). First, *Atg7^d77^* mutants showed increased oxidative stress and levels of ubiquitinated proteins, and presented defects in climbing and shortening of lifespan (Juhasz et al., 2007). Similar data were obtained with *ATG8a* mutants, while its overexpression was shown to promote neuronal survival by controlling oxidative stress and to induce longevity (Simonsen et al., 2008). Work in mice confirmed the role of ATG5 in promoting the survival of Purkinje cells and of ATG7 in preventing axonal degeneration (Komatsu et al., 2007; Nishiyama et al., 2007). Recently, GWAS studies in mice and human cells confirmed ATG5, ATG7 and identified ATG101 and ATG16L1 as crucial for maintaining neuronal survival in particular, ATG7 was associated with complex neurodevelopmental disorders in patients, confirming the role of these autophagic genes in the control of neuronal homeostasis (Wertz et al., 2020; Collier et al., 2021).

## 5. Neurodegenerative proteinopathies or (PPs)

Neurodegenerative Proteinopathies (PPs) are a large family of diseases that share common pathogenic features such as misfolded protein aggregations, neuroinflammation, oxidative stress and mitochondrial dysfunction that contribute to cellular degeneration of the neural tissue (Fleming et al., 2022). Either induced by genetic alteration or by an age-related or stochastic event, alterations of the natural conformation of proteins leads to the formation of oligomers or aggregates with consequent loss of the physiological function of the protein (Rai et al., 2022). Accumulation of aggregates in PPs is commonly due to different mechanisms that include: (i) irreversible formation of aggregates with new strong intermolecular interactions; (ii) inefficacious cell clearance; (iii) seeding and spread of pathological aggregates between cells, evident for prion-like diseases, but identified also in other proteinopathies where the presence of old aggregates function as a seed for the new one (seeds effect; Soto and Pritzkow, 2018). Thus, the central pathogenic role of protein aggregation in these diseases underlines the importance of endogenous mechanisms that control the proteostasis networks, such as the unfolded protein response (UPR), autophagy-lysosome pathway and chaperone activity (Klaips et al., 2018). In fact, age-related disruption of these proteostasis networks or their genetic alterations, contribute to the early onset of these diseases (Fleming et al., 2022; Rai et al., 2022). Deregulation of autophagy has been linked to the pathogenesis on many neuronal diseases thanks to the use of several animal models. In the current review, we will discuss only the most conserved and frequently mutated genes that are the cause of PPs (summarized in Table 1) whose characterization in flies has shown their role in protein homeostasis and autophagy, and those include HD, SCAs, ALS, AD, PD, and prion-like diseases (Pr-D).

### 5.1. Huntington’s disease

Huntington’s disease (HD) is an inherited neurodegenerative disorder caused by a dominant mutation in the first exon of the *huntingtin* (*htt*) gene that leads to an expansion of the CAG trinucleotide sequence (longer than 35 repeats), resulting in a protein with a long polyglutamine-Q stretch. The clinical onset of the disease is generally at middle age, however the length of the mutation is inversely proportional to the onset of the disease in fact, patients with long CAG show signs already at a young age (Gusella and MacDonald, 1995). The human *htt* gene encodes for a protein of 350 KDa that contains four HEAT (Huntingtin, elongation factor 3, protein phosphatase 2A and TOR kinase) domains, structurally related to the ARM (armadillo) repeats, and few PEST (peptide sequence rich in proline, glutamic acid, serine, and threonine) domains, that act as substrates for proteolytic enzymes, including caspases or calpain that cleave at amino acids 552 and 586 to produce an N-terminal fragment containing the polyQ domain (Ehrnhoefer et al., 2011). Post translational modifications (PTMs) regulates these proteolytic events and modification of the N-terminus region, such as arginine methylation (Migazzi et al., 2021; Ratovitski et al., 2022), neddylation (Ghosh and Ranjan, 2022), acetylation (Gottlieb et al., 2021), palmitoylation (Lemarie et al., 2021) and phosphorylation at Ser13 and Ser15, are relevant to modulate HTT localization and aggregate formation (Arbez et al., 2017; Chatterjee et al., 2021). These proteolytic cleavages occur also in the mutant HTT (mHTT) resulting in the formation of N-terminus truncated fragments that compete with endogenous HTT and favor the formation of toxic aggregates (Graham et al., 2006; Barbaro et al., 2015; Koyuncu et al., 2017; Ast et al., 2018). The presence of mutant HTT protein induces cellular defects leading to cell death, particularly of neurons in the striatum and cerebral cortex, leading to motor defects and rapid cognitive decline (Bates et al., 2015).

#### 5.1.1. Huntingtin and autophagy

The physical interaction between endogenous HTT and p62/SQSTM1 was shown to facilitates the binding of HTT to ULK1/Atg1 releasing its negative regulation by mTOR, thus promoting autophagy (Rui et al., 2015). The fact that HTT protein contains sequences with structural homology to Atg23, vacuole protein 8 (Vac8ar) and Atg11, containing LC3-interacting repeats (LIRs) sequences (WxxL) present only in cargo receptors suggested a role for endogenous HTT to control autophagy (Ochaba et al., 2014; Martin et al., 2015). Further data showed that myristylation at its N-terminus directed the truncated HTT to the endoplasmic reticulum to initiate autophagosome-formation (Martin et al., 2014). Conversely, the inefficient myristoylation of the mutant HTT promoted the formation of large aggregates that bind p62/SQSTM1 but were not transported to the cargo resulting in an empty autophagosome (Martinez-Vicente et al., 2010; Martin et al., 2015). The human *huntingtin* gene (*htt*) is conserved across evolution and its homologue in flies, *dhtt,* encodes for a protein that shares an overall 24% identity with the human counterpart but contains only one CAG triplet positioned at its N-terminus (Li et al., 1999). While in vertebrates the function of *htt* is essential for development and is required for neuronal survival (Nguyen et al., 2013), flies *dhtt* is not necessary for development. However, *dhtt* knockout animals exhibit a reduced Mushroom body, learning impairment, age-related impaired of motility and shortened lifespan (Zhang et al., 2009). The role of endogenous HTT in autophagy was first outlined in *dhtt* mutant animals that showed defective developmental autophagy and increased ubiquitination of p62/Ref(2)P (*Drosophila’*s p62/SQSTM1; Ochaba et al., 2014) and like its orthologue in vertebrates, Ref(2)P accumulated in brains of aged animals (Nezis et al., 2008; Bartlett et al., 2011). Furthermore, endogenous *dhtt* was shown to counteract the toxic effect of ectopic expression of the mutant human *htt-exon-1-*Q93 (Zhang et al., 2009), corroborating the protective role of endogenous HTT evidenced also in mice (Van Raamsdonk et al., 2005). More recently, loss of function mutation in the *dhtt* gene was shown to enhance defects in axon outgrowth in the Mushroom body due to decreased function of the *App* gene, the *Drosophila* orthologue of the human *amyloid beta-precursor protein* (*APP*) gene, responsible of Alzheimer’s disease, suggesting an interaction between those genes that may control the onset of AD (Marquilly et al., 2021).

#### 5.1.2. Drosophila models of HD

A few models of *Drosophila* HD have been generated over the years (Lewis and Smith, 2016). They are mostly based on the expression of the N-terminal fragments of the human *htt* gene or on the use of *htt-exon-1* mutated to carry different lengths of the CAG tract; while few models have been generated using the full-length mutant human gene due to the high toxicity of its expression when carrying the polyQ expansion tract (Romero et al., 2008). Because of their viability, flies carrying mutations in the *htt-exon-1* (mHTT) were widely used to study the function of the polyQ, and to demonstrated that the length of the CAG tract is proportional to a progressive neuronal degeneration and decline in animal motility (Jackson et al., 1998; Marsh et al., 2000; Krench and Littleton, 2017). Furthermore, expression of mHTT in neurons showed a proportional increase in the number and size of aggregates that were already visible at 48–72 h of development (Figure 1F), indicating that *Drosophila* could be an efficient model to study the kinetic and inhibitors of aggregates formation *in vivo*. Indeed, genetic screens for aggregate-phenotype modifiers, identified several pathways including TOR signaling (Ravikumar et al., 2004; Sarkar et al., 2007), the chaperons CCT (Sajjad et al., 2014; Pavel et al., 2016), histone deacetylase (Pallos et al., 2008; Jia et al., 2012), antioxidant pathways (Mason et al., 2013), deubiquitinating enzymes (Aron et al., 2018), enzymes involved in glutamine metabolism (Vernizzi et al., 2020), early endosomal protein Rab5 (Ravikumar et al., 2008), Puromycin-sensitive aminopeptidase (PSA; Menzies et al., 2010) and very recently the novel chemical compounds that function as linkers between mHTT and LC3 able to target only the mutant form of HTT to the autophagosome leaving intact the wild-type allele in a selective manner (Li et al., 2019).

### 5.2. Spinocerebellar ataxias

Spinocerebellar ataxias (SCAs) are a heterogeneous group of inherited disorders that affects the spinal cord and the cerebellum and are characterized by loss of Purkinje cells and cerebellar atrophy. SCAs present mutations in 40 different genes many of which carry an expansion of the polyglutamine tract such as SCA1, SCA2, SCA3/MJD (Machado-Joseph disease), SCA6, SCA7, SCA17 and DRPLA (Dentatorubral-pallidoluysian atrophy; Klockgether et al., 2019). Here we will focus only on the functional mechanisms characterized in *Drosophila* models for SCA1-3 (Warrick et al., 2005), since models for *CACNA1A* responsible for SCA6 (Tsou et al., 2016; Sujkowski et al., 2022), SCA7 (Jackson et al., 2005), SCA17 (Ren et al., 2011) and *DRPLA* (Charroux and Fanto, 2010) only describe motility defect in the adult flies.

#### 5.2.1. SCA1

SCA1 is an autosomal dominant disease caused by an expansion of the CAG triplet in the coding region of the *Ataxin1 (ATXN1)* gene. This gene encodes for a protein with RNA binding capacity that associate to transcriptional regulators at promoter regions (Yue et al., 2001). Patients affected by SCA1 represent 6% of the individuals affected by cerebellar ataxias, and their mutant *ATXN1* carries a tract of more than 39 *CAG* as compared to approximately 20 in the wild-type gene. The mutation leads to the production of a protein with a long polyQ that forms insoluble aggregates, visible in the nuclei of the Purkinje cells (Stoyas and La Spada, 2018). Furthermore, *ATXN1* has been shown to favor the toxicity of human pathogenic *ATXN2* in mice-models; on the contrary loss of function of *ATXN1* increased the stability of *BACE1-mRNA*, enhancing amyloidogenic cleavage of APP in a mouse model of AD (see Section 5.4.1) and outlining a function of ATXN1 also in AD (Suh et al., 2019).

##### 5.2.1.1. Ataxin1 and autophagy

A specific function for Ataxin1 in controlling the autophagic pathway in flies has not been described, however TOR inhibitors in flies were shown to ameliorate the toxic effect of mutant Ataxin1 through the release of the inhibitory role or TORC1 on the lysosomal transcription factor Mitf (*Drosophila* homologue of human TFEB; Bouche et al., 2016). *Drosophila* harbors a gene encoding for *Ataxin1* (*dAtx*) that lacks the polyQ domain, nevertheless its overexpression led to phenotypes like those obtained by human *ATXN1* overexpression, but different from those observed upon overexpression of the polyQ alone. Genetic experiments showed that Atx1 interacts through its conserved AXH domain with the zinc-finger transcription factor Senseless (Sens). This mechanism is conserved for its mammal’s homolog, the growth factor independence-1 (Gfi-1) and proposed as a potential pathogenic mechanism for SCA1 (Tsuda et al., 2005).

##### 5.2.1.2. *Drosophila* models of SCA1

Expression of the human mutant *ATXN1* gene in *Drosophila’*s neurons leads to retinal degeneration and loss of axonal projections of the interneurons in the ventral nerve cord of the brains (Fernandez-Funez et al., 2000), expression of the pathogenic form of SCA1 or SCA3 in *Drosophila* larval dendritic neurons reduced their arborization with disruption of F-actin dendritic structures, defect that is partially rescued by Rac-PAK signaling activation (Lee et al., 2011). *In vivo* screens using the adult eye helped define the role of chaperones to proteins with an expanded polyQ. In particular, the chaperone-dependent E3 ubiquitin (Ub) ligase CHIP (carboxyl terminus of Hsp70-interacting protein) and the NAD synthase nicotinamide mononucleotide adenylyl transferase (NMNAT) together with Hsp70 mediate activation of the proteosome pathway (Zhai et al., 2008). Genetic experiments have delineated the role of transglutaminases (TGs), a class of enzymes that catalyzes the formation of cross-links between glutamine residues within proteins thus increasing their stiffness and insolubility (Muma, 2007). The relevance of these enzymes in proteotoxicity and autophagy was first demonstrated using a *Drosophila* model for PD and later also in HD, where TG2 phosphorylation by PINK blocked its degradation favoring proteotoxicity and mitochondria degradation. Further experiments, both in *Drosophila* and in human cells, showed that TG2 reduction ameliorates the defects in both PD and HD (Karpuj et al., 2002; McConoughey et al., 2010; Min et al., 2015). Similarly, a role for TG5 in enhancing the toxicity of mutant *ATXN1* was shown in *Drosophila* models and in cells from SCA1-patients where TG5 was shown to colocalize with Ataxin1 (Lee et al., 2022b) further supporting a functional role for this enzymes in the disease. Genetic screen and chemical inhibitors revealed the RAS-MAPK–MSK1 as an important axis in the control of Ataxin1 aggregate formation (Park et al., 2013). Using small inhibitors of the MSK1/MAPK complex it has been demonstrated in mice and flies that the phosphorylation of Ataxin1 on Ser776 is relevant for its stability, while mutation of this residue in the wt protein reduced the level of Ataxin1-82Q in mice suggesting that Ser776 could represent a novel substrate for both drug and allele-specific therapies (Nitschke et al., 2021).

#### 5.2.2. SCA2

Mutations in the human *Ataxin2* (*ATXN2*) gene lead to spinocerebellar ataxia type 2 (SCA2) which accounts for 13% of spinocerebellar ataxias (Klockgether et al., 2019). Mutations in *ATXN2* with an expansion greater than 32 CAG repeats forms insoluble cytoplasmatic aggregates visible in Purkinje and granule cells, leading to gliosis and neuronal death (Huynh et al., 2000). Additionally, mutations in *ATXN2* were found to be a risk also for ALS due to their effect on increasing TP-43 toxicity (van den Heuvel et al., 2014).

##### 5.2.2.1. Ataxin2 and autophagy

Not many studies ruled out the function of autophagy in SCA2 until recently, when it was shown that autophagy ameliorates mitochondrial dysfunction and oxidative stress in mice models for SCA2 and in cells from patients (Wardman et al., 2020). In addition, it was shown that cordycepin, a drug that activates AMPK and autophagy, reduces the abnormal accumulation of p62/SQSTM1 and of LC3 observed in cells form SCA2 patients, indicating a dysfunction in the autophagic flux in the disease (Marcelo et al., 2021). *Drosophila* possess one orthologue of the *ATXN2* gene *(Atx2)* which encodes for a protein Atx2 that shares 23% of identity and 36% of similarity with the human Ataxin2 protein, but differently *Drosophila* Atx2 does not contain a long polyQ stretch but three short separate segments of glutamines. Structurally human Ataxin2 contains the Lilke-Sm (Lsm) and Lsm-AD domains found in RNA binding proteins (Satterfield and Pallanck, 2006), and further experiments in human and flies confirm that both Ataxin2 and Atx2 physically bind with the polyribosome suggesting that Ataxin2 may be involved in the control of translation (Satterfield et al., 2002).

##### 5.2.2.2. *Drosophila* models of SCA2

Expression of human mutant *ATXN2* in neurons resulted in the formation of aggregates leading to neuronal degeneration that (Lessing and Bonini, 2008). Further work demonstrated that overexpression of *Drosophila* Atx1 promotes the accumulation of human mutant Ataxin2 aggregates, indicating that the interaction between the two proteins could contribute to the pathogenesis of SCA1 and SCA2. This hypothesis is in line with the idea of a cooperative mechanism between Ataxin1 and Ataxin2, also suggested by the presence of Ataxin2-enriched aggregates in postmortem neurons of patients with SCA1 (Al-Ramahi et al., 2007). Further studies should be conducted using *Drosophila’s* model of SCA2 to better understand the physiological role of Ataxin2/Atx2 in the control of autophagy and how it could be perturbed in SCA2, to reveal novel mechanisms of intervention.

#### 5.2.3. SCA3

Also known as Machado-Joseph disease, SCA3 is the most common type of spinocerebellar ataxia. It is characterized by a progressive neurodegeneration caused by an expansion of CAG at the 3′ end of the coding region of the *ATXN3* gene, which from 12 to 40 repeat in the wild-type reaches more than 55 CAGs in the mutant form (Klockgether et al., 2019). The gene encodes for a deubiquitinase (DUB), a class of enzymes that counteracts the action of ubiquitin and important for the control protein stability of TP-43 and HTT (Doss-Pepe et al., 2003; van Well et al., 2019; Tran and Lee, 2022), responsible when mutated of ALS and HD, making DUBs potential targets for proteinopathies.

##### 5.2.3.1. Ataxin3 and autophagy

Autophagy is compromised by mutation of *ATXN3* since the expression of the autophagic markers p62/SQSTM1 and LC3 was found abnormal in the brain of patients with SCA3. Furthermore, transgenic mice expressing *ATXN3-71Q* exhibit large aggregates that are reduced in the presence of the autophagy-promoting gene Beclin1 (Nascimento-Ferreira et al., 2011). Puromycin-sensitive aminopeptidase (PSA), a cytosolic enzyme able to digest polyQ sequences (Menzies et al., 2010) was shown to ameliorate also Ataxin3 phenotypes both in flies and mice and to induce autophagy (Menzies et al., 2010). *Drosophila*, like mammals has two isoforms of Atx3 proteins that contain either two or three ubiquitin interacting motifs (2UIM) with an additional at its C-terminus (3UIM; Johnson et al., 2019). Studies in mammals and flies highlighted the different ability of these isoforms to form aggregates when carrying the polyQ expansion. In particular, 2UIM is more prone to form aggregates than 3UIM, that is rapidly degraded mainly through the proteasome pathway (Harris et al., 2010). Further studies in flies showed that the UIMs motifs of Atx3 interact with the heat shock protein cognate-4 (Hsc70-4) to enhance Ataxin3 mutant aggregation and toxicity (Johnson et al., 2021). This data complement those showing that the auto-protective function of *Drosophila* endogenous Atx3 depends on its deubiquitinating catalytic activity that indirectly reduce the folding of the toxic polyQ present in mutant Ataxin3 proteins as in others polyQ proteins (Warrick et al., 2005), rather than on the activation of the proteasomal degradation pathway (Tsou et al., 2015).

##### 5.2.3.2. *Drosophila* models of SCA3

The most widely used models for SCA3 express the C-terminal fragment of the human *ATXN3* gene with 78CAGs or 82CAGs repeats, that in the retinal neurons cause abnormal eye morphology due to neuronal and tissues degeneration and motility defects (Warrick et al., 1998, 2005). Genetic screens for modifiers of the Ataxin3 mutant phenotypes led to the identification of several genes of the ubiquitin pathways, members of Hsp70/chaperone proteins, and potential regulators of RAN translation, a Repeat-associated non-AUG (RAN) translation that generates toxic dipeptide repeat proteins (DPRs) from pathological repeat RNA expansions hat do not contain the classical methionine start codon (Cleary et al., 2018), see also for C9orf72-ALS (Section 5.3.1). Genetic screen in flies, using the eye, identified members of the ubiquitin ligase family, such as Cullins and of Praja1, that protect against the effect of mutant Ataxin3 strongly supporting the relevance of the ubiquitin and of the DUBs in the disease (Chen et al., 2019; Ghosh et al., 2021). This screens also identified selective modifiers of Ataxin3 able to rescue also Tau-R406W-AD mutation (Section 5.4.2) supporting the existence of common pathways that converge and contribute to neuronal degeneration, controlled by the Ataxin3 DUB-activity (Bilen and Bonini, 2007). In addition, Ataxin3 DUB-activity controls parkin’s ubiquitination in the modulation of “physiological” mitophagy (Durcan and Fon, 2011), and ubiquitination of K63 in the formation of SOD1 aggregates (Wang et al., 2012). Treatment with antioxidant drugs such as the Nrf2-inducers caffeic acid and resveratrol have also been shown to indirectly reduce apoptosis and to induce autophagy, both in human cells and in *Drosophila* models of SCA3 (Wu et al., 2017).

### 5.3. Amyotrophic lateral sclerosis

Amyotrophic lateral sclerosis (ALS) is a rare disease, with an incidence of 2–3 per 100,000 and a variable progression rate. ALS patients are diagnosed based on the degeneration of motor neurons, however, the pathophysiological heterogeneity of the disease is accompanied by a variety of other factors that are not completely understood. ALS is often associated with frontotemporal dementia (FTD), a disease that presents common mutant genes with ALS resulting in mental disability without motoneurons degeneration or movement impairment. Genetics ALS accounts for only 10–15% of all ALS cases, while the remaining are of sporadic, however up to now, more than 50 genes have been implicated in ALS and the pool of loci associated with this disease is expanding due to GWAS and whole-exome/genome sequencing. Among new pathways identified there are members of sphingolipid signaling and actin polymerization, identified in the vesicles transport network (Brenner and Freischmidt, 2022) suggesting how complex is the physiopathology of this disease. Autophagy in ALS. Deregulation of autophagy has been linked to pathogenesis of ALS thanks to the use of several animal models. ALS patients present accumulation of autophagosomes in the cytoplasm of spinal cord neurons and harbor mutations in genes involved in the autophagy machinery. Many genes have been associated to ALS and here, we will focus on C9orf72, SOD1, TD-P43, FUS since their characterization in flies has been of crucial importance to show their role in protein homeostasis and autophagy.

#### 5.3.1. C9orf72

Mutations in the *C9orf72* gene are characterized by an expansion of the hexanucleotide sequence GGGGCC (G4C2) in the first intron of the gene, which can reach up to thousands of repeats in the most affected patients (DeJesus-Hernandez et al., 2011; Renton et al., 2011). The gene encodes for a guanine nucleotide exchange involved in cellular processes such as vesicular trafficking, autophagy, lysosomal function and in the control of the immune system and recently described as part of the lysosomal membrane-complex that binds RABs for a correct lysosomal function (Taylor et al., 2016; Root et al., 2021). *C9orf72* mutation causes toxicity and alteration of the autophagy-lysosomal pathway contributing to disease pathology (Beckers et al., 2021). Hypothesis regarding the consequences of *C9orf72* mutation range from loss of gene function due to epigenetic transcriptional silencing of the locus, to RNA-mediated toxicity caused by the formation of RNA foci that trap both RNA transcripts and RNA binding proteins, and to the formation of toxic dipeptides (DPR) produced by non-AUG translation (RAN) associated with intronic hexanucleotide repeat expansion (Kwon et al., 2014; Wen et al., 2014; Freibaum and Taylor, 2017). The presence of DPRs also compromises the nucleocytoplasmic transport, resulting in the accumulation of toxic debris in the cytoplasm leading to neuronal death (Freibaum et al., 2015; Zhang et al., 2015). Here we have outlined few of these events and discussed their relationship with autophagy.

##### 5.3.1.1. *C9orf72* and autophagy

The relationship between *C9orf72* and autophagy is somewhat controversial, since some data showed that mutation in *C9orf7*2 cause toxicity and alteration of the autophagy-lysosomal pathway (Beckers et al., 2021), others showed that *C9orf72* acts as a negative regulator of autophagy as mice mutant for the *C9orf72* gene showed reduction TOR activity with nuclear translocation of TFEB and activation of the autophagy flux (Ugolino et al., 2016). Moreover, DPRs induce dysfunctions of the autophagic-lysosomal pathway by synergizing with other mechanisms of toxicity increasing the pathogenesis of the disease (Beckers et al., 2021). Growth factors and insulin/IGF influences neurodegenerative diseases including AD, PD and ALS. indeed *C9orf72-G_4_C_2_* mutation has been shown to downregulate insulin/IGF signaling in both fly and human cells while activating insulin/IGF signaling enhances the toxicity of poly-GR repeats (Atilano et al., 2021). IGFs also act as neurotrophic factors for the survival of motor neurons and have therapeutic effects in a mouse model for SOD1-ALS, and in the murine motoneurons NSC34 cells expressing mutant *C9orf72-G_4_C_2_* (Kaspar et al., 2003; Stopford et al., 2017). Using high-throughput screen to identify chemical modulators of DPRs, we also found that cells expressing mutant *C9orf72-G_4_C_2_* have increased cAMP levels resulting in protein kinase A (PKA) activation, event that was partially rescued by pharmacological or genetic inhibition of PKA (Licata et al., 2022). Since PKA is activated by growth factors, this observation raises the question of a connection between growth pathways and C9orf72 in ALS and, although the mechanisms are not yet fully understood, we presume that they may converge to the control of cellular proteostasis or autophagy.

##### 5.3.1.2. *Drosophila* models of C9orf72

Although *Drosophila* does not have a *C9orf72* orthologue, transgenic models have been established and mimic the toxicity in humans due to its gain of function effect in flies (Mori et al., 2013; Xu et al., 2013; Mizielinska et al., 2014). The insertion of repeating sequences of GGGGCCs at different lengths was used to determine their different toxicity and ability to form structures called foci that include RNA-binding proteins and RNA (Mori et al., 2013; Mizielinska et al., 2014). RNA of the DPRs obtained by RAN translation was optimized *in vitro* to express as specific peptides (poly GA, GP, GR, PA, alone or fused with GFP or FLAG tags; Cleary et al., 2018). The difference in toxicity for each DPR is related to their different chemical composition and atomic charge. Indeed, highly arginine-rich GR and PR have been shown to alter the phase separation of LCD-containing proteins by changing the charge and structure of membrane organelles and their dynamics and functions (Wen et al., 2014; Lee et al., 2016). Works in flies evidenced a direct role of *C9orf72-G_4_C_2_* in autophagy that led to the identification that its expression induced the accumulation of Ref(2)P and of poly-ubiquitinated proteins both in motor neurons and in whole larvae, due to defects in cargo protein degradation. These flies showed defects in lysosome formation, accompanied by reduced Mitf/TFEB nuclear localization (Cunningham et al., 2020). Recent work has demonstrated that nuclear transport of TFEB is mediated by nucleoporins (nuclear import proteins) and their expression is based on the molecular chaperone SIGMAR1/Sigma-1 (sigma-1 intracellular non-opioid receptor) mutated in ALS (Wang et al., 2022). Overexpression of SIGMAR1 in flies restores proper cellular transport, outlining the relevance of this chaperonin in nuclear import in this disease (Lee et al., 2020a).

#### 5.3.2. Superoxide dismutase

*Superoxide dismutase (SOD1)* is the second most frequently mutated gene in ALS, representing approximately 20% of familial ALS. This gene encodes for SOD1 a ubiquitously cytosolic Cu/Zn-binding enzyme that homodimerizes and catalyzes the dismutation of superoxide radicals to hydrogen peroxide counteracting the toxic effect of free reactive oxygen species (ROS). SOD1 modulates protein quality control, autophagy, mitochondrial function, and axonal transport. SOD1 mutations may lead to both loss or gain of function phenotypes, making difficult to interpret the mechanisms through which its mutation leads to ALS (Brenner and Freischmidt, 2022).

##### 5.3.2.1. Superoxide dismutase-ALS and autophagy

wt SOD1 was found in a complex with Atg9/Beclin1 to control P62/SQSTM1 expression, indeed mice mutant for SOD1-ALS show reduced Atg9/Beclin1 and increased level of P62/SQSTM1 and of ubiquitinated proteins (Nassif et al., 2014). Work in *Drosophila* as well as in *C. elegans*, identified a role for *lethal(2)* malignant brain tumor (L3MBTL1) a histone methyl-lysine protein, and SET domain-containing protein-8 (SETD8) in the control protein misfolding and degradation as their reduction alleviate the phenotypes induced by SOD1. This mechanism is conserved also in mammals underlining the role of chromatin modification in the control of protein quality control (Lu et al., 2019). As we know the interplay of ubiquitin-mediated autophagy is controlled also by deubiquitinases (Clague et al., 2019) and studies in *Drosophila,* as in other invertebrate models, outlined the negative role of the DUB-USP7 that by reverting the ubiquitination of the E3-ligase NEDD4L, reduced autophagy and the clearance of misfolded SOD1 and TDP-43 proteins by reducing the SMAD2/TGF-beta pathway (Zhang et al., 2020).

##### 5.3.2.2. *Drosophila* models of SOD1

*Drosophila* endogenous *dSOD1* is necessary for neuronal health as its null mutation results in neuronal loss. Co-expression of a mutant *dSOD1* allele, carrying specific structural LOF mutations, results in formation of unfunctional wt-SOD1/dSOD1 heterodimers indicating that functional dimers are necessary for the activity of the enzyme (Phillips et al., 1995). Studies in *Drosophila* and in mice demonstrated that expression of human SOD1 in motor neurons reduced ROS activity, leading to longevity (Parkes et al., 1998; Missirlis et al., 2001). Further studies in flies demonstrated that the enzymatic activity of human SOD1 is not required for its toxicity: In fact, homozygous inactive SOD1 mutants (G85R, H48R, and H71Y) expression in *dSOD1* null flies results in neurodegeneration, motor defects, and shortened life span, suggesting that these phenotypes are associated to SOD1-mutations rather than SOD1 activity (Şahin et al., 2017; Agudelo et al., 2020). Expression of mutants SOD-1 results in deposition of protein aggregates visible in neurons, glia and in skeletal muscles in both mice-of ALS and cells from ALS patients (Dirren et al., 2015). Mutations in SOD1 lead to mitochondrial impairment, a defect that also occurs in *Drosophila,* in fact mutant SOD1 accumulates in the intermembrane space of the mitochondrial membranes modifying their structure, reducing the production of ATP and causing metabolic dysfunctions particularly relevant for the activity of motor neurons (Tafuri et al., 2015; Gallart-Palau et al., 2016). A distinctive characteristic of SOD1 mutations is the presence of *non-autonomous signals from glia* that induce lethality in neighboring cells. This effect was demonstrated *in vivo* in *Drosophila* and mice models for SOD1-ALS (Boillee et al., 2006; Watson et al., 2008) and supported by data showing that ablation of the glia ameliorate the disease in mice mutants for SOD1-ALS (Guttenplan et al., 2020). The importance of non-autonomous signaling has been strengthened by recent work demonstrating that motor neurons from SOD1-G93A mutation-bearing mice exhibit metabolic changes that affect surrounding myocytes (Peggion et al., 2022), and likewise between glia and neurons to induce survival of neurons (Chua et al., 2022). Understanding the nature of the non-autonomous signals is relevant to better define the mechanisms in the pathogenesis of ALS and to identify new components and modalities of therapeutic intervention. Thus, the design of Drosophila ALS models using the combination of the two binaries, LexA-LexOp with UAS-Gal4, may allow for the expression of SOD1 mutations in a specific tissue, for example motor neurons, and the concomitant manipulation of glia, using the UAS-Gal4 where many lines are available at stock centers. This would allow to rapidly identify genes that can interfere/suppress SOD1 neuron lethality similar to the approach used for identify mHTT non-autonomous interactors (Bason et al., 2019).

#### 5.3.3. TAR DNA-binding protein 43

TAR DNA-binding protein 43 (TDP-43) is a DNA/RNA-binding protein that belongs to the heterogeneous nuclear ribonucleoprotein (hnRNP) family with both nuclear and cytoplasmic functions. Functionally, TDP-43 is involved in modulation of several aspects of RNA life such as transcription, splicing, stability and turnover, degradation, alternative polyadenylation, transport, translation, and microRNA biogenesis [165]. TDP-43 is considered a key protein in ALS for two main reasons: firstly, TDP-43 is the major component of the ubiquitin-positive cytoplasmic inclusions found in spinal motor neurons of ALS patients, secondly, mutations in its gene (*TARDBP*) occur in around 0.9% of ALS patients.

##### 5.3.3.1. TAR DNA-binding protein 43 and autophagy

Autophagy plays an important role in the clearance of the cytoplasmic inclusions in fact, it has been shown that the TDP43 aggregates colocalize with markers of autophagy and inhibition of autophagy increases their aggregates formation (Brady et al., 2011). Moreover, TDP-43 itself modulates autophagy, therefore creating a complex scenario whose perturbation contributes to ALS (Huang et al., 2020). Despite the recognized importance of TDP-43 in ALS, it is still unclear whether the pathogenesis of ALS is related to its reduced physiological function, since wild-type TDP-43 is sequestered in inclusion bodies and unable to interact with its physiological targets, or if it is due to the formation of toxic aggregates containing TDP-43 (Scotter et al., 2015). Loss of *TARDBP* in mammals downregulates histone deacetylase 6 (HDAC6), an enzyme that controls ubiquitinated protein and autophagy (Fiesel et al., 2010). Controversial results have been obtained in *TARDBP* knockout cells, where although the lysosomal transcription factor TFEB is upregulated, accumulation of immature autophagic vesicles and reduced expression of Atg7 are observed, suggesting that reduction of *TARDBP* affects other signals/genes necessary for a functional autophagy (Xia et al., 2016).

##### 5.3.3.2. *Drosophila* models of TDP-43

*Drosophila* harbors a *TARDBP* orthologue, namely *TBPH,* and several groups have investigated its function to gain insights into ALS pathogenesis by performing loss of function studies. Interestingly, *Atg7* overexpression suppresses the semi-lethality of *TBPH* null flies, ameliorating motility, lifespan and reducing the accumulation of Ref2(P)/P62 inclusions at the NMJ (Donde et al., 2020). Other studies in flies addressed the consequences of overexpressing wild-type or disease forms of TDP-43. Overexpression of a cytoplasmic wt-TDP-43 or of TDP-43-M337V mutant in the fly’s retina results in neurodegeneration that can be rescued by co-expression the heat shock protein HSP67Bc that promotes autophagy (Crippa et al., 2016). More recently, HEXA-018, a novel chemical compound that induces autophagy in a TOR-independent manner and ameliorates climbing activity and lifespan was identified as a suppressor of TDP-43 overexpression in flies (Lee et al., 2021).

#### 5.3.4. Fused in sarcoma

Fused in sarcoma (FUS) is a DNA/RNA-binding protein involved in DNA repair and RNA processing, often found in stress granules (SGs) dense structures present in the cytosol composed of RNA and RNA binding proteins (Wolozin and Ivanov, 2019). Over 70 mutations in the FUS gene have been identified in patients with familial and sporadic ALS, the vast majority of which are heterozygous mutations with autosomal dominant inheritance. Considering that most mutations influence the nuclear localization signal (NLS) of the protein, recent studies have delineated the presence of intrinsically disordered regions often enriched with arginine and glycine repeats that may be prone to promote protein aggregation, when mutated in ALS perturbating SGs dynamics that may be at the heart of ALS (Vance et al., 2009; Rhine et al., 2020). RNA binding proteins have multivalent domains important for their behavior; indeed proteins like TDP-43 and FUS, that exhibit spontaneous liquid–liquid demixing upon interaction with specific targets during phase separation, have been found to interact genetically when mutated, further accelerating neurodegeneration (Lanson et al., 2011). WT and mutant FUS are incorporated into a variety of RNA granules and they accumulate in *de novo* paraspeckles described in spinal motor neurons of ALS-patients (An et al., 2019).

##### 5.3.4.1. Autophagy and FUS

Similarly, to TP-43, autophagy seems to play a crucial positive role in the elimination of toxic aggregates also in FUS-related ALS. Expression of wild-type FUS and ALS-related FUS mutations triggers mechanisms that lead the accumulation of toxic cytoplasmic aggregates and to inhibition of autophagy (Brunet et al., 2021). Furthermore, work in flies and human iPSCs demonstrated that the P525L-FUS mutation alters stress granule dynamics resulting in inhibition of PI3K/AKT/TOR signaling which indirectly increases autophagy by a yet unknown mechanism (Marrone et al., 2018).

##### 5.3.4.2 Drosophila models of FUS

Genetic screens using flies identified Mask, an Ankyrin-repeat containing protein, that promotes the expression of the proton-pumping vacuolar (V)-type ATPases, favoring the elimination of FUS aggregates via lysosomal autophagy (Zhu et al., 2017). Recently, attention has been placed on post translational modifications that interfere with the protein phase separation of FUS, indeed the enzyme glutathione transferase omega 2 (GstO2) was shown to reduce cytoplasmic FUS level and the formation of FUS aggregates both in *Drosophila* and in mouse neuronal cells overexpressing FUS. Glutathionylation of FUS promotes its separation into the liquid phase suggesting how an accurate analysis of the mechanism driving protein phase separation might be a promising target for novel therapeutic strategies (Cha et al., 2022).

### 5.4. Alzheimer’s disease

Alzheimer’s disease (AD) is the most common form of dementia in elders (Gonzales et al., 2022), characterized by progressive neurodegeneration and cognitive impairment. This pathology is considered a proteinopathy because it is characterized by the accumulation of extracellular amyloid plaques, enriched in amyloid-β peptide (Aβ42), intracellular neurofibrillary tangles, composed of hyperphosphorylated Tau, and reactive gliosis. Most cases of AD are sporadic and only a small percentage shows clear familial autosomal dominant inheritance: the familial cases are predominantly early-onset forms associated with fully penetrant mutations in the Amyloid precursor protein (APP), and the γ-secretases Presenilin 1 and 2 (PSEN1 and 2; Karch et al., 2014). *APP* encodes for a type I transmembrane protein involved in several neuronal functions, many of which have been discovered and studied in *Drosophila* (Gunawardena and Goldstein, 2001). APP can be processed following two different pathways: the so-called amyloidogenic pathway, consisting in a subsequent action of β- and γ-secretases (BACE and PSENs) that leads to the formation of several fragments including the Aβ, and the non-amyloidogenic pathway, where the α- and γ-secretases lead to the production of different APP fragments (Vassar et al., 2009; Zhang et al., 2014). The fruit fly represents a powerful model to study AD in fact, the genes associated with AD are evolutionary conserved and models obtained by overexpression of human Aβ peptide, Tau or of APP carrying pathological mutations mimic neurodegeneration and deficit in memory/cognitive abilities.

#### 5.4.1. Amyloid precursor protein and amyloidopathies

Mutations in *APP*, *PS1*, and *PS2* genes, favor the production of the Aβ42 peptides and this, together with the presence of the amyloid plaques, shaped the “Amyloid Hypothesis” that proposes the imbalance between production and clearance of Aβ42 as the main cause of AD development (Selkoe and Hardy, 2016). To date, therapies for AD are still missing, underlying the need to better understand APP function/processing and the consequences of Aβ42 and tau aggregation.

##### 5.4.1.1 Autophagy in APP and amyloidopathies

Autophagy plays a complex and dual role in AD since it regulates both Aβ eneration and clearance (Mputhia et al., 2019). Several studies support a protective role: Atg5, Beclin1, and ULK1 were shown to be involved in Aβ degradation (Tian et al., 2011) and the adaptor protein AP2, a member of a complex responsible for the internalization of cargos in clathrin-mediated endocytosis, was shown to induce the degradation of the APP-βCTF (C-terminal fragment) via autophagy, affecting Aβ production (Tian et al., 2013). Moreover, autophagic degradation of APP-CTF and Aβ is promoted by NRBF2, a member of the class of III phosphatidylinositol 3-kinase (PtdIns3K) complex that regulates autophagosome maturation (Cai et al., 2021). On the other hand, activation of autophagy might play a detrimental role in AD depending on the context. For example, activation of TFEB, plays opposite effects on Aβ production in neurons depending on the expression level of APP or of the βCTF (Yamamoto et al., 2019; Song et al., 2020).

##### 5.4.1.2 Drosophila models of APP and amyloidopathies

*Drosophila* harbors an orthologue of *APP,* namely *APPL* and since the Aβ sequence is not conserved *Drosophila’* models of AD that contributed to gain insight into the role of autophagy were mostly obtained by overexpression of either human Aβ peptide or of human AD-associated genes. The protective action of autophagy has been proven by modulating the levels of autophagic activity in flies expressing human Aβ peptide. For example, lowering the expression of autophagy-related genes, such as *Atg1*, *Atg8a* and *Atg18*, strongly enhances the neuronal toxicity caused by Aβ expression (Omata et al., 2014). In line with this, *Atg8a* overexpression in an Alzheimer *Drosophila* model obtained by overexpression of human amyloid precursor (hAPP) and beta-secretase 1 (hBACE1), enhances stress-resistance, slows down neurodegeneration and improves lifespan (Tsakiri et al., 2021). In a similar AD model, promotion of autophagy by the Nicotinamide mononucleotide adenylyltransferase (NMNAT) reduced amyloid plaques formation (Zhu et al., 2022). In flies overexpressing Aβ42, the protective role of autophagy was demonstrated by the expression of Thioredoxin-80 (Trx80), which prevents the accumulation of the toxic peptides in the brain and rescues lifespan by inducing autophagosome formation (Gerenu et al., 2021). On the other hand, Ling and colleagues showed that dysfunctional AEL (autophagy-endosomal-lysosomal) plays a crucial role in Aβ42 accumulation. In fact, the abnormal accumulation of Aβ42 within AEL vesicles was reduced when the functional autophagy was decreased (modulation of *Atg5* or *Atg12* levels), suggesting that dysfunctional autophagy–endosomal–lysosomal may cause Amyloid-like plaque formation (Ling et al., 2014). Moreover, it was recently shown that ectopic APP expression is sufficient to trigger aberrant autophagy through its interaction with the carboxyl-terminus of Hsc70-interacting protein (CHIP), a U-box type chaperone associated E3 ubiquitin ligase via transcriptional upregulation of *BACE1* and *PSEN* inducing an AD-like neurodegeneration (Zhuang et al., 2020).

#### 5.4.2. Tau and tauopathies

Tau is a group of six microtubule-associated proteins all derived from alternative splicing of the *MAP/Tau* gene, they are abundant in the CNS, where they are necessary for the stabilization of the microtubules important for axonal transport. In the presence of specific mutations or during aging, Tau becomes hyperphosphorylated and changes its structure, decreases its ability to bind microtubules, leading to the formation of insoluble filaments that accumulate as neurofibrillary tangles resulting in tauopathies, a hallmark of AD (Goedert et al., 2017; Hernandez et al., 2022). Recent work pointed to a role of Tau within the nucleus where it associates with nucleolar proteins, suggesting the presence of other mechanisms activated by mutation of Tau (Anton-Fernandez et al., 2022).

##### 5.4.2.1. Autophagy in tauopathies

Autophagy plays a protective role in tauopathies, as it favors the clearance of the soluble and the insoluble Tau form the last present in the aggregates. Furthermore, autophagy helps the movement of Tau through vesicles involved in its transport into neurons.

##### 5.4.2.2. *Drosophila* models of tauopathies

*Drosophila* possesses a *tau* gene with 46% identity and 66% similarity to the corresponding human *Tau*. Flies have been successfully used to pin down numerous pathways that control Tau toxicity via autophagy. For example, insulin signaling can induce abnormal Tau phosphorylation and accumulation, inhibiting its autophagic clearance and worsening the disease (Chatterjee et al., 2019). Silencing calpain, a family of proteases that inhibit autophagy, is protective against Tau toxicity in the *Drosophila-*eye, an effect that requires a functional autophagy since it was lost in *Atg8a* mutant flies (Menzies et al., 2015). A microRNAs screen aimed at identifying suppressors of Tau-mediated phenotypes, clarified that *CG11070*, a target of miR-9a mediates Tau ubiquitination and degradation primarily via the autophagy-lysosome system. Interestingly, this function is conserved for the human orthologue *UBE4B* in cells of neuroblastoma (Subramanian et al., 2021). In AD patients Tau protein is often hyper-phosphorylated and one of the kinases responsible is PTK2/FAK (focal adhesion kinase; Lee et al., 2022a). PTK2 plays a critical role in proteinopathies as it favors abnormal phosphorylation of proteins, including TDP-43 and p62/SQSTM1 leading to the formation of ubiquitinated aggregates and neurotoxicity induced by the dysregulation of the ubiquitin–proteasome system (UPS; Lee et al., 2020b). This mechanism is conserved for Tau (wt or mutant P301L) and also present in *Drosophila* where it was shown that attenuation of PTK2 expression reduces the phosphorylation of Ref2(P)/p62 ameliorating the motility of Tau expressing flies (Lee et al., 2022a). This also suggests the presence of a feed-back loop between PTK2 and p62 dysregulated in tauopathies.

### 5.5. Parkinson’s disease

Parkinson’s disease (PD) is the second most common neurodegenerative disorder after Alzheimer’s disease and another example of tauopathy (Poewe et al., 2017). PD symptoms include slow movement, postural imbalances, resting tremor, and muscle rigidity. Non-motor symptoms are also common and include memory loss, psychiatric symptoms, sleep problems, and pain. These symptoms stem from dopaminergic (DA) neuron loss in a part of the brain known as the substantia nigra pars compacta (SN), which leads to a lack of dopamine in other regions of the brain including the striatum. Both the affected regions are involved in movement and muscle control (Maiti et al., 2017). Treatment with the dopamine precursor levodopa or drugs such as dopamine agonists can temporarily improve many of the motor symptoms of PD however, no treatment currently exist able to prevent the neurodegenerative processes, underlying the importance to study the mechanisms responsible for DA neuron loss (Poewe et al., 2017). PD is characterized by the presence of Lewy Bodies, cytoplasmic inclusions consisting of a variety of misfolded proteins including α-synuclein and phosphorylated tau, in the SN and throughout the brain. As for Alzheimer’s Disease, most cases of PD are sporadic, thought to be triggered by environmental factors. However inherited forms of PD, linked to mutations in several different genes including *α-synuclein, LRRK2, GBA, Parkin, PINK1*, and *Vps35* do exist, and their pathology and progression is very similar or indistinguishable from the sporadic one.

#### 5.5.1 Autophagy in AD

The misfolded proteins accumulated in the Lewy Bodies are thought to be toxic, triggering neuroinflammation and cell death (Lashuel et al., 2013), and even though the exact mechanisms leading to their accumulation and toxicity are not completely understood, it seems that they include mitochondrial dysfunction, oxidative stress, and disruption of autophagy-mediated protein clearance (Karabiyik et al., 2017; Maiti et al., 2017). Interestingly, most of the genes associated with PD, such as *Parkin, PINK1,* and *LRRK2*, control mitochondria activity and are involved in autophagy-mediated degradation/ubiquitination of proteins to control the turnover of damaged mitochondria (mitophagy; Winklhofer, 2014; Rahman and Morrison, 2019). Most of the PD-associated genes are expressed and functionally conserved in *Drosophila*, therefore both loss of function of endogenous genes or models harboring different human mutations were generated (Hewitt and Whitworth, 2017). Work performed in the fruit fly has largely contributed to clarifying the role of these genes in autophagy/mitophagy. In the following paragraphs, we will discuss the most common *Drosophila* models of PD used to study autophagy modulation in this disease.

#### 5.5.2. Alpha-synuclein (α-syn)

The *SNCA* gene encodes for α-synuclein (α-syn), a protein that is abundantly expressed in the nervous system where it is found in membranes and is a major components of the Lewy Bodies. Mutations in α-syn have been identified in both familial and sporadic cases of PD (Lesage and Brice, 2009). While some carry single-amino acid substitutions that increased α-synuclein secretion (Guan et al., 2020), most mutations promote α-syn aggregation and fibrils formation (i.e., E46K, H50Q, A53T), while others (i.e., G51D and A53E) decelerate α-syn aggregation (Flagmeier et al., 2016; Guan et al., 2020). This suggests that the effects of PD-associated pathogenic mutations on α-syn behavior are quite complex and probably modulated by different signaling.

##### 5.5.2.1. *Drosophila* models of α-synuclein

Flies do not possess an orthologue of *α-syn* therefore models have been produced by ectopically expressing human mutants of *α-syn*. A large part of studies conducted in flies point to a dysfunction of autophagy in presence of *α-syn* mutation and to a positive role of autophagy in α-syn induced degeneration: ectopic expression of human α-syn leads to F-actin-mediated impairment in autophagic flux, accumulation of abnormal autophagosomes, impairment in mitophagy, leading to defects in cellular bioenergetics (Sarkar et al., 2021). Treatment of flies overexpressing α-syn with spermidine, a naturally occurring polyamine known to prolong lifespan by inducing autophagy, protects against α-syn induced neurotoxicity (Buttner et al., 2014). Overexpression of the human lysosomal membrane protein LAMP2A fully prevents the behavioral defects induced by neuronal expression of a PD-associated mutant form of α-syn (SNCA/A30P) as well as reduces the α-syn accumulation in older flies. Moreover, LAMP2A expression upregulates Atg5 that stimulates macroautophagy (Issa et al., 2018). Similarly, the progressive locomotor decline and the loss of dopaminergic (DA) neurons caused by the human α-syn-A30P variant is reduced by pan-neuronal overexpression of dDOR (orthologue of the human tumor protein 53-induced nuclear protein 1 (TP53INP1), able to activate autophagy and basal mitophagy (Dinh et al., 2021). Another group demonstrated that knockdown of inositol-requiring enzyme 1 (IRE1) or Atg7 reverses α-syn-A30P-induced neurodegeneration in terms of lifespan, locomotor activity, and DA neuron loss (Yan et al., 2019). This suggests that IRE1 and Atg7 may play different roles in the classical autophagy mechanism of survival and their loss activates protective mechanisms thereby explaining the reduction of toxic proteins and neuron survival in PD.

#### 5.5.3. Parkin/Pink1

One of the molecular characteristics of PD are defects in mitophagy, the process that allows the elimination of damaged mitochondria, and this leads to accelerated neurodegeneration.

##### 5.5.3.1. *Drosophila* models of Parkin/Pink1

Parkin and Pink1 are involved in mitochondria quality control and turnover (Shimura et al., 2000), the recruitment of the E3 ubiquitin ligase Parkin on the damaged mitochondrial membrane by the kinase Pink1, controls mitochondrial turnover by inducing a physiological level of mitophagy (Truban et al., 2017). *Drosophila* harbors only one *parkin* and *PINK1* orthologue (Clark et al., 2006) and their function is highly conserved. Several models have been created both using endogenous *parkin* and *PINK1* null mutants and by overexpressing human genes harboring pathogenic mutations, that closely recapitulate many PD features including DA loss, decreased lifespan, and motor defects (Ishihara-Paul et al., 2008; Hewitt and Whitworth, 2017). In particular, these models have been fundamental to understand the molecular mechanisms linking parkin and Pink1 to mitophagy. Studies using flies’ flight muscles identified their relevance in the control physiological mitophagy (Greene et al., 2003), furthermore, new studies outline how the increase in mitophagy observed in aging depends on the parkin/Pink1 interaction and on the activity of two deubiquitinases USP15 and 30 (Cornelissen et al., 2018). The mitochondria phenotype observed in *parkin* null mutants recapitulates general autophagy inhibition obtained by loss of *Atg7*, supporting the physiological role of parkin in the activation of mitophagy as clearance pathway (Vincow et al., 2013). Many proteins have been identified that may affect parkin/Pink1 modulation of mitophagy: Fbxo7, whose gene *PARK15* is mutated in early-onset autosomal recessive forms of Parkinson, directly interacts with parkin to control its translocation to the mitochondrial membrane and functionally interacts with Pink1 to regulate parkin-dependent mitophagy (Burchell et al., 2013). The mitochondrial protein BNIP3L, a BH3 protein member of the Bcl2 family, interacts with Pink1 to induce mitophagy, and work in flies’ muscles helped to understand the role of human BNIP3 as suppressor of Pink1-induced-mitophagy (Zhang et al., 2016). BNIP3L was identified as a substrate for PARKIN2, and this ubiquitination recruits the autophagic cargo receptor NBR1 to the mitochondria to induce PARKIN2-mediated mitophagy. Even if PARKIN2 is present only in mammals, it was demonstrated using *Drosophila* that ectopic expression of BNIP3L rescues the mitochondrial defects of *PINK1* mutant but not the effect of *parkin* mutant, outlining that PARKIN2 is the principal substrate for BNIP3L and it is necessary for BNIP3L clearance of mitochondria (Gao et al., 2015). Using flies, it was also demonstrated how the reduction of the human orthologue of Mask (ANKHD1), a scaffolding protein that inhibits mitophagy, rescues *parkin/PINK1* mutant defects, suggesting that the human ANKHD1 might represent an interesting novel therapeutic target for treating PD associated to parkin/Pink1 mutations (Zhu et al., 2015). A novel mechanistic link between mitophagy and translation lies in the evolutionarily conserved function of EFTU, a mitochondrial translation elongation factor, that acts as substrate and interacts with Pink1 independently of parkin. Pink1 regulates mitophagy by restraining EFTU in the cytosol after phosphorylation on Ser222, inhibiting mitophagy by interacting with the Atg5-Atg12 complex formation (Lin et al., 2020). Despite several studies strongly linking parkin/Pink1 to mitophagy, Lee et al. (2018) demonstrated that basal mitophagy is not strongly reduced in the muscles of *parkin/PINK1* mutant animals, and similar results were obtained using mouse models for PD under conditions of high metabolic demand. A possible explanation could be that the metabolic state of cells could influence mitophagy, particularly in animals carrying *parkin/PINK1* mutations (McWilliams et al., 2018).

#### 5.5.4. Leucine rich repeat kinase-2

Leucine rich repeat kinase-2 (LRRK2) is a leucine-rich repeat cytoplasmic kinase found also on the mitochondrial outer membrane, which contains a central core with a ROC-GTPase and other protein–protein interaction domains, suggesting multi-functional activity. LRRK2 is involved in several signaling pathways including vesicle trafficking, mitochondrial function, autophagic and lysosomal pathways, protein translation, neurite outgrowth, and cytoskeletal arrangement (Cookson, 2010). Mutations in LRRK2 are the most common genetic cause of both familial (41%) and sporadic (1–3%) PD (Jia et al., 2022; Oun et al., 2022) and among the function of LRRK2 that have been already clarified, it seems that its role in endomembrane trafficking may play a role in the pathogenesis of PD (Roosen and Cookson, 2016). Notably, recent experiments in human cells, also demonstrated that LRRK2 reduction is protective against the formation of α-syn aggregates suggesting a common signaling between these proteins. These studies pointed to glial cell function, in which the LRRK2 mutation interferes with the ability of glia to clear neuronal α-syn aggregates with a mechanism that is still unclear. The relevance of astrocytes in PD and proteinopathies is an important topic that needs attention (see Section 6) as more and more data delineate the presence/relevance of non-autonomous signals between astrocytes and neurons critical for neuronal clearance and survival (Streubel-Gallasch et al., 2021).

##### 5.5.4.1. *Drosophila* models of LRRK2

*Drosophila* harbors a single *LRRK2* orthologue gene (*dLRKK*) therefore it has been used to better understand the physiological functions of LRRK2 using loss of function models or overexpression of human *LRRK2* variants responsible for PD (Hewitt and Whitworth, 2017; Seegobin et al., 2020). Overexpression of pathogenic mutant forms of *LRRK2* causes defects in endolysosomal and autophagic assembly, and LRRK2-targeted therapies in PD are based on this molecular event. However, several studies in *Drosophila* have shown that *dLRKK* reduction affects lysosomal compartments and leads to the accumulation of dysfunctional autophagosomes, suggesting that proper expression of LRRK2 is required for functional signaling of autophagy and vesicle trafficking (Dodson et al., 2014; Seegobin et al., 2020). LRRK2 controls the phosphorylation of several small GTPases of the Ras-associated binding protein (Rab) family responsible for vesicle trafficking at the synapse level (Steger et al., 2016; Bellucci et al., 2022). Another relevant observation is the interaction of LRRK2 with other proteins leading to PD. Indeed, Ng and colleagues demonstrated that overexpression of the disease-associated LRRK2/G2019S mutant in flies flight muscles induces mitochondrial pathology and impairs locomotion by phenocopying the behavior of *parkin*-null flies, whereas in DA neurons it leads to significantly enlarged mitochondria. In both cases, the toxic effects of LRKK2 mutants were rescued by co-expression of parkin or activation of AMPK, known to reduce TOR signaling and to activate autophagy (Ng et al., 2012), suggesting a cooperative function in mitochondria between LRRK2 and parkin.

### 5.6. Prion diseases

Prion diseases (PrD) are a group of disorders characterized by the presence of aberrantly shaped proteins, called prions (*proteinaceous infectious particles*) encoded by the *PRNP* gene, that cause the accumulation of misfolded aggregated proteins in the central nervous system (Geschwind, 2015). Some forms occur sporadically, others are inherited or acquired through and include Creutzfeldt-Jacob disease (CJD), Gerstmann-Straussler-Scheinker syndrome (GSS), Fatal Familial Insomnia (FFI), and Kuru disease. Their characteristic is to induce transmissible spongiform encephalopathies (TSE) which lead to inflammation and neuronal death, with symptoms and severity depending on the type of prion and the animal species (Geschwind, 2015).

#### 5.6.1 Autophagy in PrD

PrP^C^, responsible for this class of diseases, is an abundant protein inserted into the cell membrane via a GPI domain located at its C-terminus while the N-terminal tail contains functional glycosylation sites (Scheckel and Aguzzi, 2018). PrP^C^ undergoes conformational changes transforming into an infectious misfolded isoform, named scrapie-associated prion protein (PrP^Sc^) responsible for the toxic effect of the infection (Sandberg et al., 2011). *PrD and autophagy*. Proteostasis and autophagy play a central role in protecting neurons from the toxic effect of PrP^Sc^ (Thellung et al., 2022). Several studies have outlined a direct role of PrP^C^ in the control of autophagy: in fact, the reduction of PrP^C^ impairs the cellular response to oxidative stress with consequent dysregulation of the autophagic flux. Conversely, ectopic expression of PrP^C^ appears to have a protective role by inducing autophagy under stressful conditions (Jeong and Park, 2015). Inducing autophagy is beneficial for the disease indeed, treating mice expressing the infective prions with rapamycin prolong their survival, while in yeast the induction of autophagy reduces the formation of prion protein PrP^Sc^ (Speldewinde et al., 2015). Similarly, transcriptomic analysis using the *Drosophila* model of PrP^Sc^ revealed a perturbation in genes component of TOR signaling suggesting a role in the control of translation by mutant PrP^Sc^ (Thackray et al., 2020). Furthermore, in the brains of prion-infected rodents the level of p62/SQSTM1 increases and co-localizes with PrP^Sc^ in large aggregates surrounding the perinuclear region of the cells (Homma et al., 2014). This mechanism resembles that observed in HD, where p62/SQSTM1 accumulates in the perinuclear area due to the formation of large ubiquitinated aggregates thus blocking its ability to transport cargo proteins into the autophagosome. However, the ubiquitin-binding protein p62/SQSTM1 not only mediates autophagy but also controls the activation of the Nuclear factor erythroid 2 (Nrf2), a key regulator of the cellular antioxidant response (Bellezza et al., 2018; Gureev et al., 2020). Thus inactivation of p62/SQSTM1 in prion diseases may contribute to the higher oxidative stress present in the cells (Shah et al., 2018). Recently, p62/SQSTM1 was found in cytoplasmic inclusions together with PrP^C^ and the E3-ligase/TRAF6. Moreover, its activity was required for proper redistribution of PrP^C^ into the insoluble cell fraction suggesting a novel physiological interaction between p62/SQSTM1 and PrP^C^ (Masperone et al., 2022). Recent evidence suggested that neurodegeneration in prion diseases may be the consequence of defective glia as astrocytes have been shown to deposit mutant PrP aggregates and propagate prions to neurons and other cells (seed effect; Tahir et al., 2022). To this end we would like to suggest that *Drosophila* could be an optimal model to start exploring the contribution of glia to neuronal survival/death during prion pathogenesis using the combination of binary systems (see Sections 2 and 6).

#### 5.6.2 Drosophila models of PrD

*Drosophila models of PrD*. *Drosophila* genome does not contain a *PrP* orthologue, making flies ideal for identifying the gain-of-function mechanisms associated with PrP misfolding (Fernandez-Funez et al., 2017). Common PrP mutations were first created in *Drosophila* using a mutation found in the *CJD-Prp-PG14* family with nine additional repeats (Deleault et al., 2003). *Prp-PG14* was expressed in neurons of the brain, in photoreceptors and pigmented cells of the eye but surprisingly, Prp-PG14 did not accumulate in the neurons and flies showed no behavioral or neuropathological abnormalities. In contrast, the expression of *Prp-P101L* (the murine orthologue of human *PrP-P102L*) resulted in locomotor defects and accumulation of PrP-P101L aggregates and vacuolation similar to what observed in neurons of GSS patients (Gavin et al., 2006). Surprisingly, using an inducible system it was shown that PrP-GSS phenotypes could be reverted and its level decreased when its expression was blocked, representing the first demonstration of reversibility of a phenotype reported in a genetic model for prion disease (Murali et al., 2014). *Drosophila* is also susceptible to exogenous sources of prions, indeed, it has been shown that PrP toxic phenotype can be transferred from fly to fly and from mice to fly. In these experiments, *Drosophila* larvae expressing variants of the *PrP^WT^* or carrying the toxic *PrP^3F4^* mutant epitope were exposed to a homogenate of mouse or *Drosophila* brains expressing amino acid substitutions associated with the human prion diseases *FFI* and *CJD* (Thackray et al., 2017). These experiments demonstrated that both *Drosophila* adult-animals *PrP^WT^* or *PrP^3F4^* exposed to prions show a significant decline in locomotor ability and of survival, indicating that the cellular and molecular components for prion replication and toxicity, were present and functional also in flies (Thackray et al., 2017). Furthermore, transcriptomic analysis using a *Drosophila* model of mutant PrP revealed a perturbation in cell cycle genes, regulator of protein synthesis and mitochondrial function, revealing data very similar to those in mammalian hosts undergoing to prion disease, further supporting the idea that flies are a well-established animal model to study mammalian prion biology (Thackray et al., 2020). More recently, new transgenic flies expressing different mutations of PrP in neurons highlight the function of PERK (EIF2AK3 in humans) and of the activating transcription factor 4 (ATF4), members of the UPR response in mediating PrP toxicity. A partially protective activity was shown for 4E-BP (EIF4EBP2 in humans), in the disease induced by human PrP-M129 and PrP-V129 mutations, on the contrary mutations in human PrP-(N159D, D167S, N174S) revealed a partial reduction of the toxic activity induced by co-expression of N159 mutation (Myers et al., 2022), highlighting how *Drosophila* can be used to study important amino acid substitutions to link PrP structural propensities to its toxicity.

## 6. Why using *Drosophila* to study PPs

### 6.1. To study non autonomous glial-neuron signaling *in vivo*

Cell-autonomous degeneration of neurons was considered the main outcome of neurodegenerative proteinopathies until several studies highlighted the causal role of glia and the relevance of non-cell autonomous signals that may influence neuronal health (Hickman et al., 2018; Yamanaka and Komine, 2018; Acioglu et al., 2021). Glial cells play important physiological functions in the CNS trough non-autonomous signals such as release of small molecules necessary for neuronal survival or inducing toxicity causing their death (Acioglu et al., 2021; Goodman and Bellen, 2022). The exploitation of cell type-specific expression systems, such as UAS-Gal4 and LexA-LexOp, would make fruit flies a suitable model to study the non-autonomous interactions between cells (see section 2). Indeed, it has been demonstrated that non-cell autonomous mechanisms play a key role in mutation for SOD1 (ALS), LKRR2 (PD) and in HD (Van Harten et al., 2021), with mechanisms not totally clear. Phagocytic glia may be activated by the dying neurons promoting their clearance but this may also worsen the disease as glia releases inflammatory neurotoxic cytokines toxic for the cells as shown for HD (Crotti et al., 2014; Creus-Muncunill and Ehrlich, 2019). A non-autonomous mechanisms is activated by the expression in glia of the chaperon DNAJB6, ortholog of the human HSP-DNAJ, that protects neuronal degeneration and extend lifespan (Bason et al., 2019), A new component is the Triggering *Receptor* Expressed on Myeloid cells (TREM2), a microglial phagocytic transmembrane receptor, conserved also in flies, for which genetic variations have been associated with senile dementia and increased risk of AD (Filipello et al., 2022). Data in mammals showed that its N-terminal cleavage blocks amyloid-β oligomerization in neurons exerting a protective role in AD. TREM2 also enhances glia metabolism and its function in clearance (Filipello et al., 2022); we may speculate that in AD TREM soluble-extracellular domain may act non-autonomously to activate its protecting signals on Aβ oligomerization.

### 6.2. To study protein-aggregate formation *in vivo*

Aggregates can be visualized *ex vivo* in organs (brain, muscles) either by immunofluorescence or by using the gene expressed as fusion protein with fluorescent proteins. The size and number of aggregates can be easily quantified with imaging techniques and applied to visualize changes upon performing: edible screens using chemical libraries; genetic screens for interactors or to identify signals, i.e., between glia and neurons that modify aggregates formation and dimensions. Glia-mediated phagocytosis might also favor prion-like seeding mechanisms characteristic of some PPs that drive aggregate formation (Pearce et al., 2015; Soto and Pritzkow, 2018; Donnelly et al., 2022). The seed-mechanism occurs also in flies and it was demonstrated in a model for HD using FRET/FRASE where the migration of mHTT aggregates was monitored *in vivo* in fly brains, and it has been proposed that glia phagocytosis might be involved in the rapid grow of mHTT aggregates *in vivo* (Ast et al., 2018). This observation is corroborated by a new mechanism in yeast showing that polyQ oligomers forms “*de novo*” aggregates and increase their original size by directly using the endogenous prion-forming protein Rnq1 in its amyloid-like prion conformation (Gropp et al., 2022).

### 6.3. To study the cross-talks of genes acting in the same proteinopathies

As we have seen, mutations in some PP can affect other genes suggesting common pathways to be analyzed. For example, Dewan and colleagues identified pathogenic HTT repeat expansions in patients diagnosed with FTD/ALS neurodegenerative disorders with mutations in *TDRPH* (Dewan et al., 2021), while polyQ expansions in the *ATXN2* gene, that are normally associated with the onset of SCA2, have been observed in some forms of ALS (van den Heuvel et al., 2014). In addition, increasing evidence points to prion-like transmission as a mechanism underlying the development of many proteinopathies, driven by α-syn, HTT, SOD1, Tau, TDP-43, which can be uptake by the cytoplasm of acceptor cells from the brain (i.e., phagocytic microglia; Bayer, 2015; Pearce et al., 2015; Jo et al., 2020; Holec et al., 2022; Trist et al., 2022). Again *Drosophila* models can be easily engineered to co-express different mutations and their cross-talks with specific pathways such as autophagy or cell death, or their response to specific chemical drugs, can be easily analyzed *in vivo*.

### 6.4. To identify biomarkers

Nowadays much attention is paid to the identification of biomarkers for an early diagnosis/detection of proteinopathies (Doroszkiewicz et al., 2022). To analyze the direct secretion of non-autonomous signals (see section 6.1), or conveyed by Extracellular Vesicles (EV; Quiroz-Baez et al., 2020; Picca et al., 2022) or by exosomes (Beatriz et al., 2021; Zhang et al., 2021; Kumari and Anji, 2022) could identify molecules that may have a pathological role in the diseases (Jackson et al., 2022). In *Drosophila,* markers are available to identify EVs or exosomes, this, together with the advantage of generating a large progeny and the use of advanced biochemical tools, could accelerate this area of intervention.

In conclusion, the field of proteinopathies is in continuous evolution and there is still much to uncover about the misfolding process that is at the basis of the protein accumulation and disease propagation. *Drosophila* represents a powerful tool to dissect the many aspects that we still need to understand and represent an important alternative to the use of other model organisms. For example, the short time frame needed for the formation of the toxic aggregates in flies is an incredible advantage when studying the dynamic of aggregates formation upon modulation of signaling pathways or with chemical compounds. These data can be complemented with those obtained directly from drug screen using 3Dculture of patients-derived organoids rather than using 1D tissue culture lines. *Drosophila* can also by-pass mouse models for proteinopathies in the initial phase of characterization of novel human genes whose function is still unknown, using genetic screens. However, there are also some pitfalls to consider since the lack of an immune system and of proper microglia limits a deeper understanding of the physiology of the PPs. Nevertheless, we would like to underline the relevance of implementing *Drosophila* studies of the function of glia in PPs since it is more targetable for therapies than neurons, and because many data suggest the presence of non-autonomous cellular mechanisms, that are difficult to address in the available mammalian models.

## Author contributions

SS, CL, CC, LT, and LV wrote and revised the review. PB and AS organized the topics and wrote and revised the review. All authors contributed to the article and approved the submitted version.

## Funding

5 × 1000 tax campaigned for the research on neurodegenerative diseases (University of Trento).

## Conflict of interest

The authors declare that the research was conducted in the absence of any commercial or financial relationships that could be construed as a potential conflict of interest.

## Publisher’s note

All claims expressed in this article are solely those of the authors and do not necessarily represent those of their affiliated organizations, or those of the publisher, the editors and the reviewers. Any product that may be evaluated in this article, or claim that may be made by its manufacturer, is not guaranteed or endorsed by the publisher.
